# Identification of oral cancer related candidate genes by integrating protein-protein interactions, gene ontology, pathway analysis and immunohistochemistry

**DOI:** 10.1038/s41598-017-02522-5

**Published:** 2017-05-30

**Authors:** Ravindra Kumar, Sabindra K. Samal, Samapika Routray, Rupesh Dash, Anshuman Dixit

**Affiliations:** 1 Institute of Life Sciences, Nalco Square, Bhubaneswar, 751023 Odisha India; 2 Manipal University, Manipal, 576104 Karnataka India; 3 Siksha ‘O’ Anusandhan University, Bhubaneswar, 751003 Odisha India; 4 All India Institute of Medical Sciences, Sijhua, Bhubaneswar, 751019 Odisha India

**Keywords:** Gene ontology, Network topology

## Abstract

In the recent years, bioinformatics methods have been reported with a high degree of success for candidate gene identification. In this milieu, we have used an integrated bioinformatics approach assimilating information from gene ontologies (GO), protein–protein interaction (PPI) and network analysis to predict candidate genes related to oral squamous cell carcinoma (OSCC). A total of 40973 PPIs were considered for 4704 cancer-related genes to construct human cancer gene network (HCGN). The importance of each node was measured in HCGN by ten different centrality measures. We have shown that the top ranking genes are related to a significantly higher number of diseases as compared to other genes in HCGN. A total of 39 candidate oral cancer target genes were predicted by combining top ranked genes and the genes corresponding to significantly enriched oral cancer related GO terms. Initial verification using literature and available experimental data indicated that 29 genes were related with OSCC. A detailed pathway analysis led us to propose a role for the selected candidate genes in the invasion and metastasis in OSCC. We further validated our predictions using immunohistochemistry (IHC) and found that the gene FLNA was upregulated while the genes ARRB1 and HTT were downregulated in the OSCC tissue samples.

## Introduction

Head and neck cancers are among the 10 most common cancers globally^1^. Oral squamous cell carcinoma (OSCC) in males is the most common cancer amongst all head and neck squamous cell carcinoma (HNSCC). It includes approximately 90% of oral malignancies and accounts for more than 300,000 of newly diagnosed cases every year^2^. It is estimated that the number of HNSCC cases will increase to 1 million by the year 2030 (http://www.who.int/entity/healthinfo/statistics/bod_dalybywhoregion.xls?ua=1). Despite significant progress in cancer treatment and management, the mortality rate associated with OSCC remains unchanged. The molecular basis of aggressive OSCC growth and metastasis is still unclear. The OSCC often goes undetected until advanced stages which are related to high mortality rate, and as such it carries heavy personal and societal costs. The overall 5-year survival rate is estimated about 50%^3^. Therefore, new targets for early diagnosis of OSCC and its treatment are required to reduce the mortality due to oral cancer.

The identification of the genes associated with complex diseases has become an important task in recent years. Experimental approaches, e.g. genetic linkage association studies^4^, expression profiling^5^, and genome-wide association studies^6^ have been found to be successful in identifying high relative risk genes for diseases like cancer^7^, asthma^8^, diabetes^9^ and hypertension^10^. However, the disease heterogeneity, complexity in finding the gene in a given locus and the cost involved led to the development of various *in-silico* approaches for disease gene identification. These approaches usually involve data mining^11^, analysis of biological sequences, phenotype^12^ and expression data^5^, protein-protein interactions (PPI)^11,13–15^, gene ontology (GO)^13,15^, gene regulatory networks and pathways for identification of candidate gene(s)^5,11,13^. Many of the recent publications have reported the successful use of such techniques for prioritizing disease-related genes and their drug targets^5,13^.

Direct PPIs are one of the strongest indications of a functional relation between genes and this information can be utilized for the identification of candidate genes^12^. The PPI networks can be constructed using physical interactions, functional and expression data, etc. Physical interactions provide the information about how two proteins are connected to each other. These interactions regulate various biological activities like transcription, translation, and degradation of RNAs and proteins. These interactions can be represented as a network graph where a product like protein is a node or vertex, and their relation e.g. physical interaction is a link or edge. Such networks can be of two types on the basis of edges (i) directed: where the relation is directional and (ii) undirected: where the direction of the relation is not considered. The PPI networks have been utilized for candidate gene identification in many studies^12,14,15^. The networks can also be classified by their topological properties as scale-free and small world networks, where the node degree distribution follows the Power law or Poisson distribution respectively. The biological networks are generally scale-free networks i.e. they are characterized by the presence of many hubs that have hundreds of interactions. The PPI data can be used in combination with GO for identification of disease-associated genes^13,15,16^. The GO term is summarized as a relationship between a gene and biological function. It provides a common vocabulary to describe aspects of a gene product’s biology, allowing genes from different species to be compared based on their GO annotations. There are three types of GO terms viz. biological process (BP), molecular function (MF) and cellular component (CC). The BP is defined as a complete process that contains a set of molecular functions like replication or cell cycle. The MF is a specific activity at molecular levels such as binding or catalysis, whereas CC represents the part of a cell or its extracellular environment.

In the last few years, it has been realized that networks in natural, technological and social systems follow a series of basic organizing principles in their structure and evolution that distinguish them from randomly linked networks^17^. The biological network based approaches have been used to identify pathways related to diseases and the candidate gene(s) related to particular pathways. Gilman *et al*. reported the identification of autism affected genes network using network-based analysis of genetic association^18^. PPI network in combination with GO has been used to predict the genes associated with immunodeficiency^14^, systemic lupus erythematosus^13^, and cervical cancer^16^. Recently, Jin *et al*. have reported the identification of a twelve gene network module based on differential co-expression PPI network in ovarian serous cystadenocarcinoma^19^.

**Figure 1:**
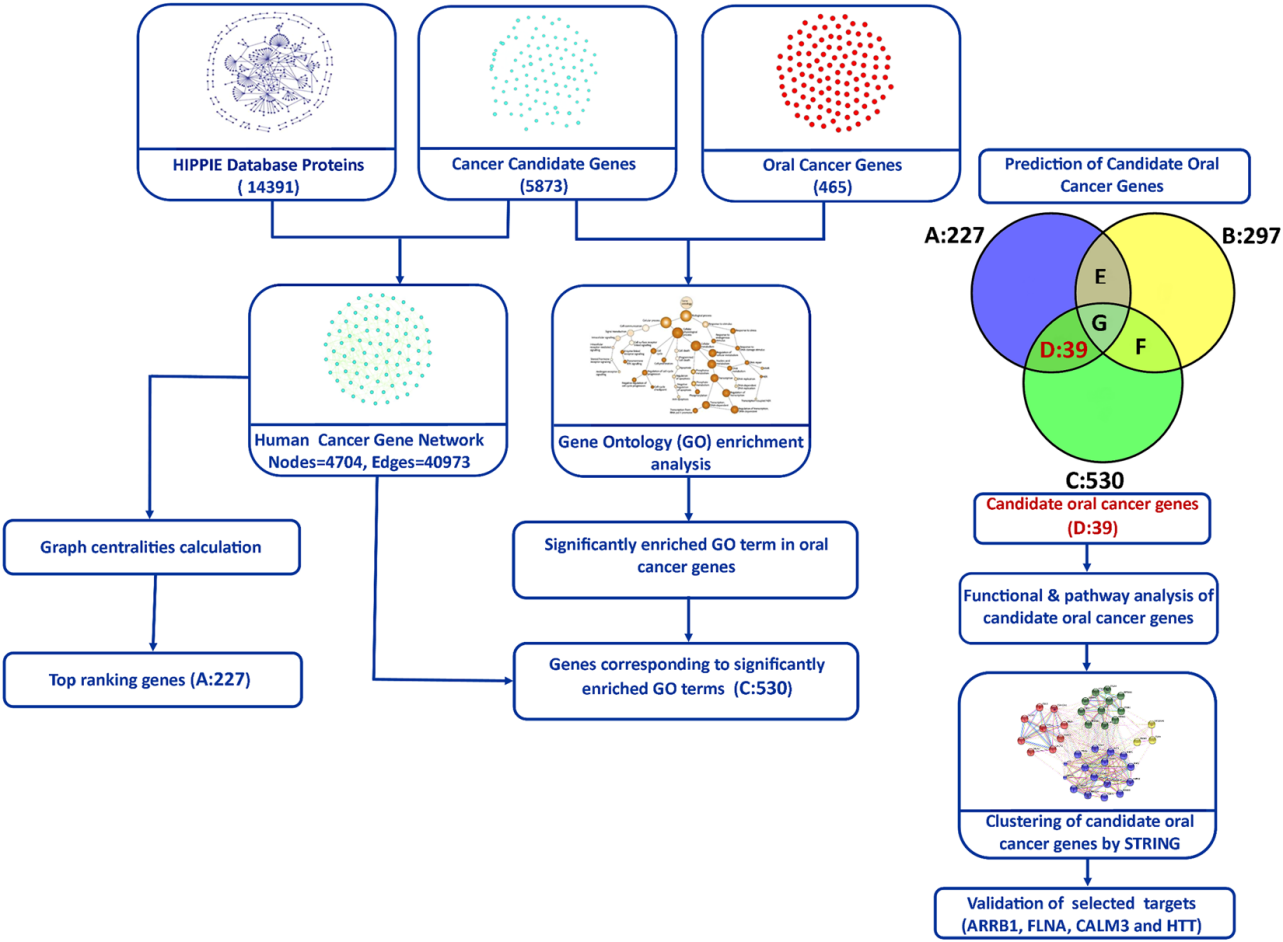
The flowchart of the methodology. The data sources (HIPPIE, Cancer candidate genes and Oral cancer genes) are indicated on the top. The HCGN was created from HIPPIE interactions in cancer candidate genes. The top ranking genes in HCGN (Set A, 227 genes) were identified using a combination of centralities. The genes common between oral cancer genes (465) and genes in HCGN (4704) were defined as oral cancer genes in HCGN (Set B, 297 genes). The GO enrichment was done using oral cancer genes and cancer candidate genes. The genes corresponding to significantly enriched GO terms were identified (Set C, 530 genes). The candidate oral cancer genes (Set D, 39 genes) were predicted using Set A, Set B and Set C. The Set E and F contains oral cancer genes in HCGN that are either top ranked or corresponding to enriched GO terms respectively. Set G contains the oral cancer genes in HCGN that are both top ranked and corresponding to enriched GO terms. A pathway and cluster analysis was done on predicted genes (Set D) and selected candidates were validated.

In the current work, we have used an integrated approach that integrates information from various sources such as experimental data, web resources, and PPIs to identify candidate genes involved in OSCC. The identified genes were then validated using literature survey, online experimental data and immunohistochemistry (IHC) from patient samples (Fig. 1).

## Results

The top ranked 5% genes from consensus ranking list (Set A), oral cancer genes in HCGN (Set B) and genes corresponding to disease associated enriched GO terms (Set C) were analyzed to predict novel oral cancer related genes.

### The human cancer gene network

The cancer candidate genes were obtained from The Cancer Gene Atlas (TCGA)-generanker (total 5873 candidate genes) (http://cbio.mskcc.org/tcga-generanker/sources.jsp). These were mapped to HIPPIE (Human Integrated Protein-Protein Interaction rEference) database v1.7^20^. This database contains 193486 interactions among 15519 proteins (v1.7, 2014). There are 15383 interactions in this database that are coming from literature only (no experimental evidence). In the current study, we have used HIPPIE score cutoff of 0.63 to select more reliable interactions that resulted in the exclusion of almost 1/4th of (44779) the PPI interactions. Among the remaining 148707 interactions, there were only four interactions that are inferred from literature only.

**Figure 2:**
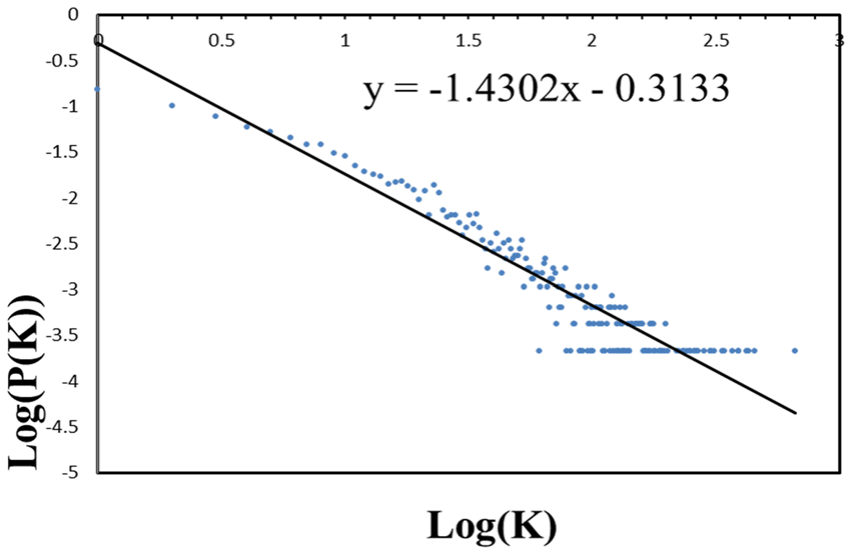
The graph of degree distribution for HCGN, where K is the degree and P (K) is the fraction of nodes in the network with degree K. The exponent value of 1.4302 indicates that the network is a scale-free network.

The network of cancer genes was created using the remaining PPIs (148707). Out of 5873 cancer candidate genes, a total of 4759 genes were mapped to 14391 proteins containing 40977 interactions. The largest interconnected component of the network was then selected by removing parallel interactions, loop and the orphan nodes. This resulted in a network containing 4704 nodes and 40973 interactions that was defined as human cancer gene network (HCGN) (Fig. 1). It is also important to note that there is no edge in the HCGN that is coming solely from the literature. The topological parameters like average degree, graph diameter, average distance, graph density, modularity, global efficiency and average clustering coefficient were calculated to characterize the HCGN and found to be 17.420, 8.000, 3.174, 0.004, 0.341, 0.322 and 0.180 respectively. The degree distribution of HCGN follows the Power law distribution with exponent value of 1.416 indicating the network is a scale-free network (Fig. 2). The scale-free networks are usually natural networks that are dominated by hubs. These networks are also vulnerable to specific alterations. The HCGN has more than three hundred highly connected nodes with a degree of 50 or higher. The highly connected proteins with a central role in the network’s architecture are more likely to be essential than other proteins^21,22^. The average degree of HCGN was found to be 17.42, indicating that the majority of proteins are highly connected. Thus, there are greater chances of having conserved PPIs. It has to be noted that essential PPIs are more conserved than nonessential PPIs^23^. The highly connected proteins can be lethal, if by some alterations they become hyper/hypo functional^21^.

### HCGN graph centralities calculation and ranking

A total of eleven different centralities were calculated for the nodes of HCGN. The genes were then ranked by individual centrality scores. A total number of unique rankings by each centrality are given in Supplementary Table S1. The data indicates that some of the centralities e.g. centroid, degree and closeness may not be able to give good distinction amongst nodes as the number of unique scores given by them are quite less as compared to the number of nodes in the network (Supplementary Table S1). The vulnerability gave the highest number of unique scores which may result in better distinction among nodes.

The pair-wise rank correlation was also calculated for the ranks given by the centralities to the genes (Supplementary Table S2). The rank correlation coefficient among the centralities shows that the centralities are highly inter-correlated. Some of the centralities have 100% correlation (Supplementary Table S2), thus only one out of them was taken for further study. Therefore, total ten centralities were considered for final analysis. It is interesting to note that there was a good agreement among centralities for rankings of the genes i.e. a gene ranked higher by one centrality was usually ranked similar by other centrality (Supplementary Table S3).

Oral cancer related genes were collected from two databases viz. Head-Neck and Oral Cancer Database (HNOCDB) (http://gyanxet.com/hno.html) and Oral Cancer Gene Database (OrCGDB) v2 (http://www.actrec.gov.in/OCDB/index.htm) containing 465 oral cancer genes together. It was found that there are 297 genes common between 465 oral cancer genes and 4704 HCGN genes. These genes were defined as oral cancer genes in HCGN (Set B). The distribution of these genes (set B) was checked in top 2%, 5%, 7% and 10% ranked genes by each centrality to select a cut-off for prediction of the candidate genes (Table 1). The unique pooled genes contain the unique genes from all centralities combined together at a particular cutoff e.g. 2%. Additionally, a consensus ranking was calculated for each gene by summing up all ranks given by different centralities. The precision score for top 2%, 5%, 7%, and 10% of the consensus ranked genes was found to be 30.77, 24.67, 23.85 and 19.87 respectively (Table 1). This indicates that consensus ranking is clearly better than individual centralities or unique pooled genes (Table 1). The precision decreases as we move from top ranked 2% to top ranked 10% genes. Though the top ranked 2% genes comparatively showed greater fraction of oral cancer genes in HCGN, the total number of unique oral cancer genes was very less (28 out of total 91 genes, Table 1). Therefore, to make a balance between precision and number of known oral cancer genes we have decided for a cutoff of top 5% ranked genes (56 out of 227(Set A) genes, Table 1) for downstream analyses.

**Table 1:** The distribution of oral cancer genes in HCGN in top ranked lists.

	Centrality	Degree	Closeness	Centroid	SPB	Eigen vector	Page rank	CFC	CFB	Stress	Vulnerability	Unique pooled genes	Consensus Ranking
**Top 2%**	Oral cancer genes^#^	2	22	18	24	26	13	27	21	23	21	37	28
	Total genes	4	67	46	76	90	47	90	71	74	84	152	91
	Precision*	50.00	32.84	39.13	31.58	28.89	27.66	30.00	29.58	31.08	25.00	24.34	30.77
**Top 5%**	Oral cancer genes^#^	5	41	31	43	46	32	51	43	44	45	71	56
	Total genes	9	170	109	193	221	121	221	181	180	211	359	227
	Precision*	55.56	24.12	28.44	22.28	20.81	26.45	23.08	23.76	24.44	21.33	19.77	24.67
**Top 7%**	Oral cancer genes^#^	8	55	37	59	68	42	65	56	56	58	94	57
	Total genes	13	237	157	269	309	168	308	252	251	295	517	239
	Precision*	61.54	23.21	23.57	21.93	22.01	25.00	21.10	22.22	22.31	19.66	18.18	23.85
**Top 10%**	Oral cancer genes^#^	8	75	52	77	85	53	83	71	73	70	118	89
	Total genes	18	354	230	383	442	239	440	359	357	423	762	448
	Precision*	44.44	21.19	22.61	20.10	19.23	22.18	18.86	19.78	20.45	16.55	15.48	19.87

*Precision is the percentage of oral cancer genes in total genes. ^#^Common out of 297 oral cancer genes in HCGN.

### GO enrichment analysis

The GO enrichment analysis for oral cancer genes was performed using GORILLA (Gene Ontology enRIchment anaLysis and visuaLizAtion tool) (http://cbl-gorilla.cs.technion.ac.il/) in two list mode where the target was oral cancer genes (465) and the background was cancer genes (6023). The GO enrichment analysis resulted in the identification of 903 enriched GO terms (785 BP, 52 MF and 66 CC). However, this is a large number and may result in the prediction of a vast number of genes where many of them could be false positives. Therefore, a criterion is needed to reduce the total number of GO terms to increase the efficiency of prediction.

In some of the reported studies, the GO terms which were associated with less than 3 and more than 50 genes (3 < = B < = 50, B = number of genes) were filtered out to remove non-specific terms^13,16^. In our case, this method gave total 227 enriched GO terms including BP, MF and CC. We found 935 genes in HCGN corresponding to these GO terms. Out of these, 188 (~20%) were found to be common with oral cancer genes in HCGN. However, this criteria was arbitrary and may result in higher noise.

In the current study, two additional criteria were tested (i) top 20% enriched GO terms (ii) top 20% enriched GO terms with the removal of redundant GO terms by REVIGO (http://revigo.irb.hr/), to select significantly enriched GO terms (Table 2). The number of top ranked 20% enriched GO terms was found to be 172 that was reduced to 103 GO terms (i.e. a reduction of ~40%) after removal of redundancy. The total number of significantly enriched GO terms (BP) were 85. The highly enriched terms includes GO:0031340 (positive regulation of vesicle fusion) and GO:0043353 (enucleate erythrocyte differentiation). There were nine significantly enriched GO terms (MF) which include GO:0008379 (thioredoxin peroxidase activity) and GO:0008191 (metalloendopeptidase inhibitor activity). Similarly, in case of CC there were nine significantly enriched GO terms which includes GO:0001533 (cornified envelope), and GO:0045095 (keratin filament). The details are shown in Supplementary Table S4. Corresponding to these 103 GO terms, there were 530 genes (including 152 oral cancer related genes) in HCGN. These 530 genes were used for the prediction of oral cancer related genes.

**Table 2:** Analysis of different criteria to select specific GO terms.

S. No.	Criteria	Number of oral cancer genes in HCGN	Total genes	*Precision
**1**.	3 < = B < = 50	188	935	20.10
**2**.	Top 20% enriched GO terms	170	684	24.85
**3**.	Top 20% enriched GO terms and redundancy removal	152	530	28.67

*Percentage of oral cancer genes in total genes.

### Prediction of oral cancer candidate genes

#### The three gene sub-sets were defined as follows

**Figure 3:**
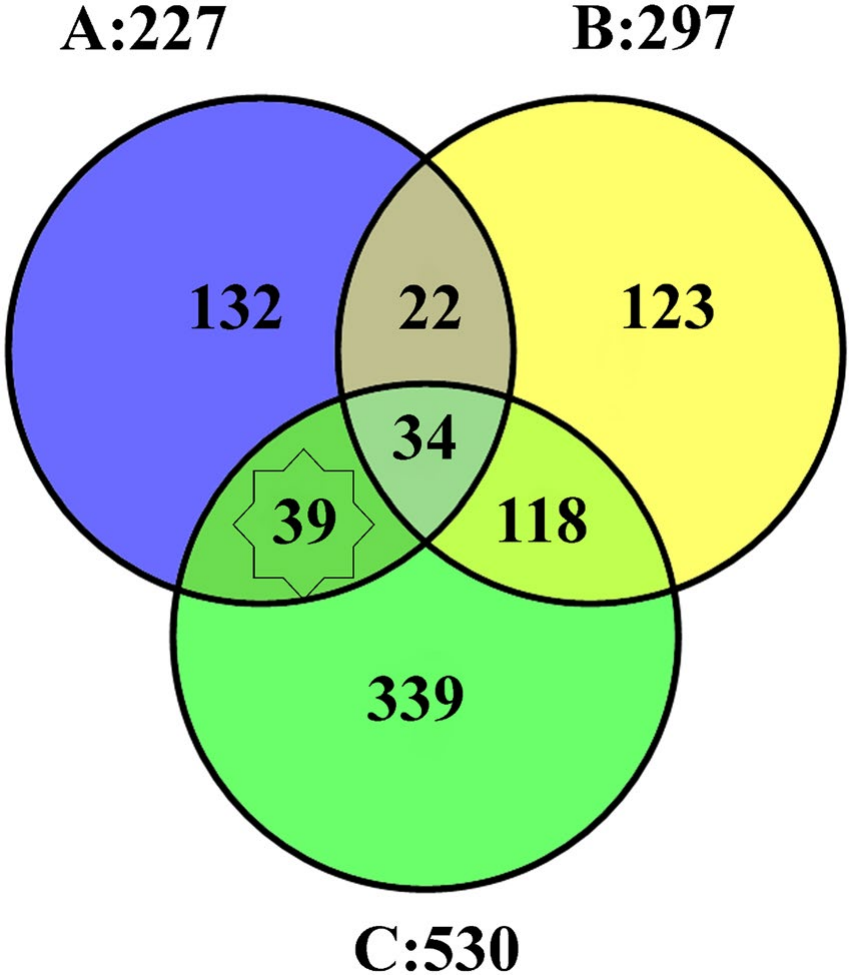
Prediction of candidate genes using top ranked genes (Set A), oral cancer genes in HCGN (Set B) and genes corresponding to significantly enriched GO terms (Set C). The genes shared by Set A and Set C but not by Set B were predicted as candidate genes.

Set A contains top 5% consensus ranking genes (227 genes), Set B contains oral cancer genes in HCGN (297 genes common between all oral cancer genes 465 and HCGN genes 4704) and Set C contains genes associated with significantly enriched GO terms (530 genes). These set of genes were compared to predict the potential candidate genes for oral cancer (Fig. 3. Briefly, the genes that were present in Set A and Set C but not in Set B were defined as candidate genes). Set B was not considered for target prediction as these genes were already given in known datasets for oral cancer. This method resulted in the prediction of 39 potential candidate genes (Table 3). These genes were taken for downstream analysis.

**Table 3:** The candidate oral cancer genes.

S. No.	Gene ID	Gene Symbol	HCGN Ranking	Degree with known oral cancer genes	Expression Atlas (mRNA)	Oncomine mRNA expression*
**1**	3320	HSP90AA1	1	55		Up
**2**	8452	CUL3	2	59		NA
**3**	2099	ESR1	3	41	Up	Down
**4**	3312	HSPA8	4	30	Up	Up
**5**	1499	CTNNB1	5	32		NA
**6**	6714	SRC	6	0		NA
**7**	4088	SMAD3	7	20	Up	NA
**8**	5071	PARK2	8	21	Down	Down
**9**	5970	RELA	9	23		NA
**10**	207	AKT1	10	28		NA
**11**	408	ARRB1	11	18		NA
**12**	7186	TRAF2	12	21		NA
**13**	1457	CSNK2A1	13	25		Up
**14**	7013	TERF1	14	15		Up
**15**	5591	PRKDC	15	25		Up
**16**	3064	HTT	15	15		NA
**17**	23411	SIRT1	17	20		NA
**18**	5518	PPP2R1A	18	14		NA
**19**	10014	HDAC5	19	21		NA
**20**	4176	MCM7	20	21		Up
**21**	3676	ITGA4	21	38		Up
**22**	1432	MAPK14	22	15		NA
**23**	808	CALM3	23	12		NA
**24**	805	CALM2	23	12		NA
**25**	801	CALM1	23	12		NA
**26**	4851	NOTCH1	26	10		NA
**27**	5515	PPP2CA	27	16		Up
**28**	2316	FLNA	28	14	Up	Up
**29**	5894	RAF1	29	19		NA
**30**	8841	HDAC3	30	11		NA
**31**	8826	IQGAP1	31	12	Down	NA
**32**	5747	PTK2	32	23		Up
**33**	6657	SOX2	33	7	Down	NA
**34**	6195	RPS6KA1	34	9		NA
**35**	5580	PRKCD	35	15		NA
**36**	3717	JAK2	36	15		NA
**37**	998	CDC42	37	5		Up
**38**	6709	SPTAN1	38	12		NA
**39**	3688	ITGB1	36	14	Down	NA

*The mRNA expression status (oral cancer vs normal) of a gene as reported in the Oncomine database and expression ATLAS. NA = Not reported.

### Evidence in literature/databases for predicted candidate genes

It is always pertinent to verify the *in-silico* predictions. In this study, a validation can be done by looking at differential gene expression in normal vs. oral cancer samples.

The predicted genes (total 39) were checked for their differential mRNA expression in oral cancer samples in the Oncomine database (https://www.oncomine.org) and the Expression Atlas (https://www.ebi.ac.uk/gxa/home). The carcinomas of tongue, floor of the mouth and oral cavity were considered for the current study. A gene was considered differentially expressed, if the fold change was greater than 2 and the p-value was less than 0.01. In the Oncomine database, a total of thirteen predicted genes were found to have differential expression in oral cancer tissues (Table 3 and Supplementary Table S5). Among them, eleven genes were found to have higher expression in oral cancer samples. The genes PRKCD and MCM7 were found to be highly upregulated as evidenced by fold change of 7.29 and 5.94 respectively. On the other hand, two genes (PARK2 in tongue and ESR1 in oral cavity) were found as downregulated with the fold change of 2.98 and 3.59 respectively. The Expression Atlas showed 8 of the 39 genes to be differentially expressed in OSCC samples (4 upregulated and 4 downregulated) (Table 3 and Supplementary Table S5**)**.

Further, a detailed literature survey was carried out for the 39 predicted genes and it was found that 24 genes have literature related to oral cancer. The genes CTNNB1 and MCM7 have been well studied for oral cancer but were not present in the databases which were taken initially for this study. It was interesting to see these genes in the predictions. Taken together Oncomine, Expression Atlas and literature, 29 out of predicted 39 genes have shown association with oral cancer which clearly indicates the robustness of the present method. Ten genes (CUL3, HTT, ARRB1, TERF2, PPP2R1A, PRKCD, RPS6KA1, HDAC5, HDAC3 and SPTAN1) were not found to have any association with oral cancer in literature and oncomine data. The detailed literature information of all 29 genes is given in supplementary information (Supplementary Table S6).

### Pathway and functional analysis

**Figure 4:**
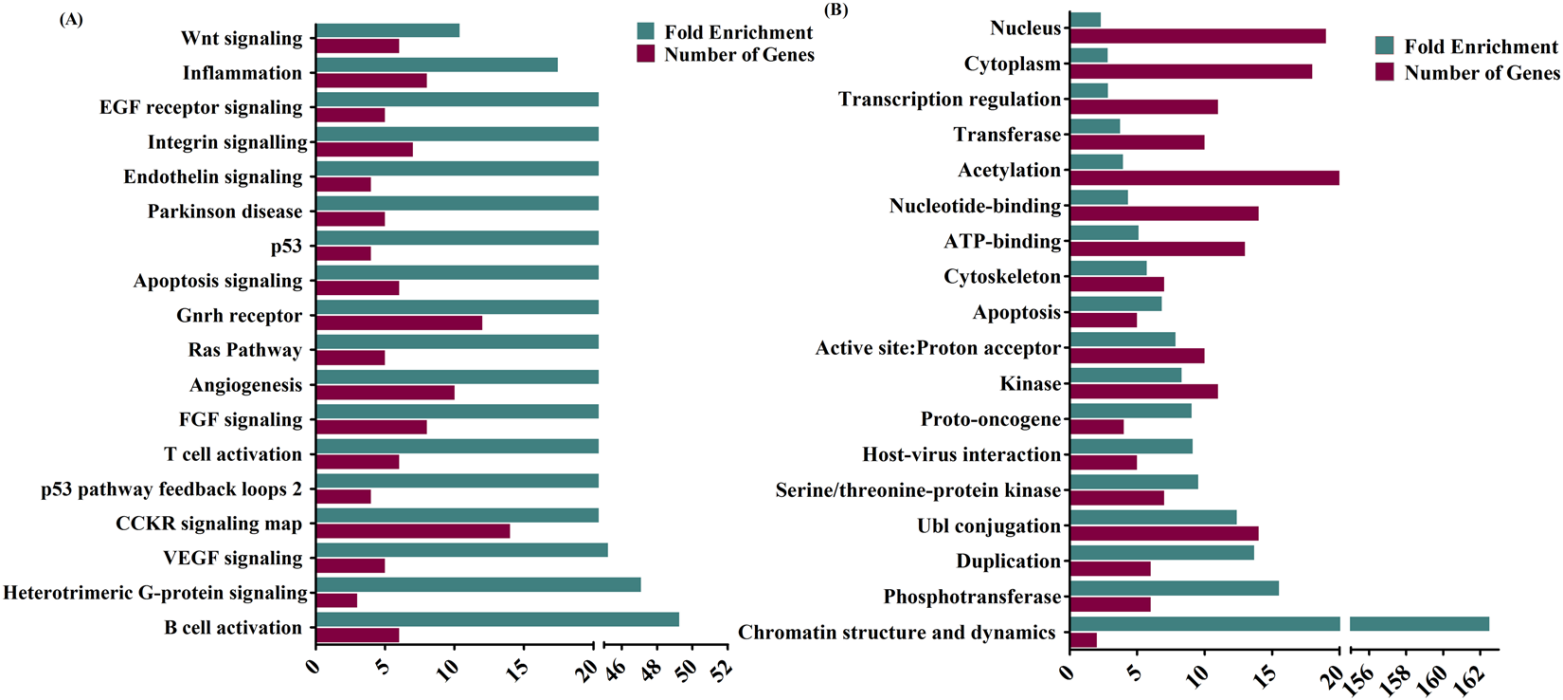
The enriched pathways (**A**) and functions (**B**) corresponding to their number of genes and fold change.

The pathway analysis was performed for the predicted 39 genes using PANTHER (Protein ANalysis THrough Evolutionary Relationships)^24^. A total of eighteen pathways were found to be significantly enriched (p-value < 0.01) (Table 4**)**. The cholecystokinin receptor (CCKR) signaling and gonadotropin releasing hormone receptor pathway were found to have the majority of predicted genes (~36% and ~ 31% respectively). It is imperative to note that many of the genes are common in different pathways i.e. they overlap among pathways. However, there are no reports of involvement of these pathways in the pathology of OSCC. The B cell activation and heterotrimeric G-protein signaling pathway-rod outer segment emerged as two most highly enriched pathways (Fig. 4A). Both of these pathways have already been reported in the context of OSCC^25,26^.

**Table 4:** The pathway enrichment analysis.

Enriched Pathways	Genes*
B cell activation	CALM1, CALM2, CALM3, MAPK14, PRKCD, raf1
Heterotrimeric G-protein signaling pathway-rod outer segment phototransduction	CALM1, CALM2, CALM3
VEGF signaling pathway	AKT1, MAPK14, PRKCD, PTK2, RAF1
p53 pathway feedback loops 2	AKT1, CTNNB1, MAPK14, PPP2CA
CCKR signaling map	AKT1, CALM1, CALM2, CALM3, CDC42, CTNNB1, ITGB1, JAK2, MAPK14, PRKCD, PTK2, RAF2, RPS6KA1, SRC
T cell activation	AKT1, CALM1, CALM2, CALM3, CDC42, RAF1
Ras Pathway	AKT1, CDC42, MAPK14, RAF1, RPS6KA1
Angiogenesis	AKT1, CTNNB1, ITGA4, ITGB1, MAPK14, NOTCH1, PRKCD, PTK2, RAF1, SRC
FGF signaling pathway	AKT1, ITGA4, ITGB1, MAPK14, PPP2CA, PPP2R1A, PRKCD, RAF1
Apoptosis signaling pathway	AKT1, HSPA8, PRKCD, RELA, SP101, TRAF2
p53 pathway	AKT1, PP2CA, SIRT1, TRAF2
Gonadotropin releasing hormone receptor pathway	AKT1, CDC42, CTNNB1, HTT, ITGB1, MAPK14, PRKCD, PTK2, RAF1, RELA, SMAD3, SRC
Parkinson disease	CSNK2A1, HSPA8, MAPK14, PARK2, SRC
Endothelin signaling pathway	AKT1, ARRB1, PARKCD, RAF1
EGF receptor signaling pathway	AKT1, MAPK14, PPP2CA, PARKCD, RAF1
Integrin signalling pathway	CDC42, FLNA, ITGA4, ITGB1, PTK2, RAF1, SRC
Inflammation mediated by chemokine and cytokine signaling pathway	AKT1, ARRB1, ITGA4, ITGB1, JAK2, PARKCD, RAF1, RELA

*The predicted genes related to the corresponding pathway.

The predicted genes (39) were classified into eighteen different functional classes with the significant p-value < 0.01 using DAVID (Database for Annotation, Visualization and Integrated Discovery) (Table 5)^27^. The function class “chromatin structure and dynamics/secondary metabolites biosynthesis, transport and catabolism”, which includes genes HDAC5 and HDAC3, was found to be highly enriched. The majority of genes were classified in a nucleus (~51%), acetylation (~54%) and cytoplasm (49%) (Fig. 4B). Many of the genes were classified in cytoplasm and nucleus simultaneously such as MAPK14, ARRB1 and SMAD3.

**Table 5:** The functional enrichment analysis.

Enriched Functions	Genes
Acetylation	TRAF2, PPP2R1A, HSP90AA1, RELA, SMAD3, PRKDC, SIRT1, ITGB1, FLNA, IQGAP1, HDAC5, CDC42, PTK2, CSNK2A1, MCM7, PPP2CA, CALM3, CALM2, HSPA8, CALM1, SPTAN1, TERF1
Active site:Proton acceptor	AKT1, PTK2, CSNK2A1, RPS6KA1, MAPK14, RAF1, JAK2, SIRT1, PRKCD, SRC
Apoptosis	AKT1, TRAF2, HTT, SIRT1, CTNNB1
ATP-binding	AKT1, PTK2, CSNK2A1, HSP90AA1, MCM7, RPS6KA1, MAPK14, PRKDC, RAF1, JAK2, PRKCD, SRC, HSPA8
Chromatin structure and dynamics/Secondary metabolites biosynthesis, transport, and catabolism	HDAC5, HDAC3
Cytoplasm	TRAF2, HSP90AA1, HTT, RELA, SMAD3, PARK2, PRKCD, FLNA, CTNNB1, AKT1, HDAC5, ARRB1, MAPK14, PPP2CA, CALM3, CALM2, HSPA8, CALM1, TERF1, SPTAN1
Cytoskeleton	PPP2CA, CALM3, ITGA4, CALM2, FLNA, SPTAN1, TERF1, CALM1, CTNNB1
Duplication	CALM3, ITGA4, ITGB1, PRKCD, CALM2, IQGAP1, FLNA, CALM1
Host-virus interaction	RELA, SIRT1, ITGB1, SRC, HSPA8
Kinase	AKT1, PTK2, CSNK2A1, RPS6KA1, MAPK14, CALM3, PRKDC, RAF1, JAK2, PRKCD, CALM2, SRC, CALM1
Nucleotide-binding	AKT1, CDC42, PTK2, CSNK2A1, HSP90AA1, MCM7, RPS6KA1, MAPK14, PRKDC, RAF1, JAK2, PRKCD, SRC, HSPA8
Nucleus	HTT, RELA, SOX2, ESR1, SMAD3, PRKDC, PARK2, SIRT1, CTNNB1, CUL3, AKT1, HDAC5, HDAC3, NOTCH1, MCM7, ARRB1, MAPK14, PPP2CA, TERF1
Phosphotransferase	CSNK2A1, RPS6KA1, MAPK14, PRKDC, PRKCD, SRC
Proto-oncogene	AKT1, RAF1, JAK2, SRC
Serine/threonine-protein kinase	AKT1, CSNK2A1, RPS6KA1, MAPK14, PRKDC, RAF1, PRKCD
Transcription regulation	HDAC5, HDAC3, NOTCH1, MCM7, ARRB1, RELA, SOX2, ESR1, SMAD3, SIRT1, CTNNB1
Transferase	AKT1, PTK2, CSNK2A1, RPS6KA1, MAPK14, PRKDC, RAF1, JAK2, PRKCD, SRC
Ubl conjugation	TRAF2, HTT, RELA, SOX2, ESR1, PARK2, CTNNB1, CUL3, AKT1, HDAC5, HDAC3, ARRB1, CALM3, CALM2, CALM1, TERF1

*The predicted genes related to the corresponding function.

### Identification of clusters in the predicted genes

**Figure 5:**
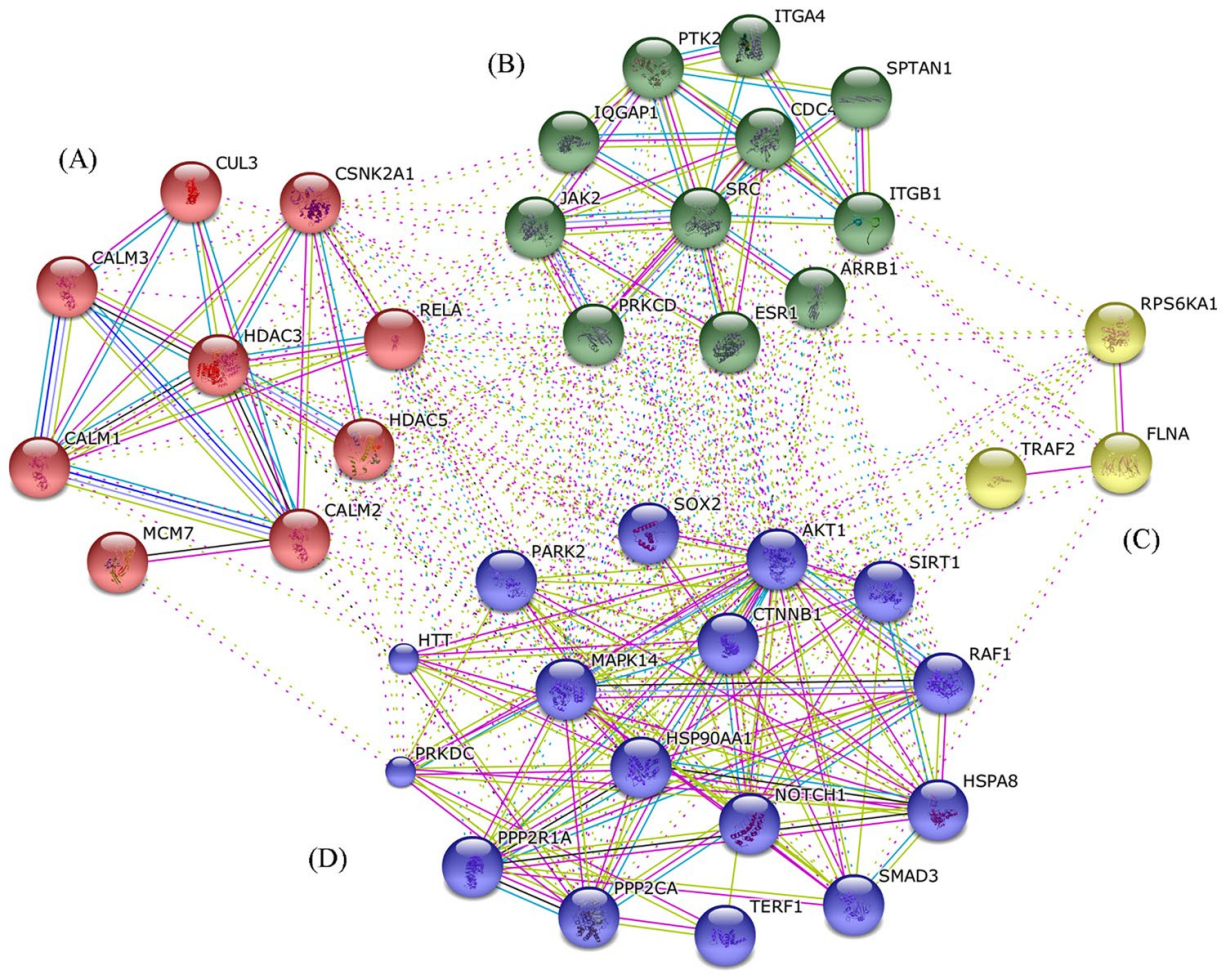
The cluster analysis of predicted 39 genes using STRING. The identified clusters are colored in red (**A**), green (**B**), yellow (**C**) and blue (**D**). The solid and the dotted lines indicate connection within the same and different cluster respectively. Different color indicates different type of interactions. (Cyan-from curated databases; Pink-experimentally determined; Blue-gene co-occurrence; Khaki-from text mining; Black-co-expression; Light blue-protein homology).

The clustering is one of the first steps in computational genetics analysis. The cluster analysis was performed on the predicted 39 genes using the K-means^28^ clustering method in STRING (Search Tool for the Retrieval of Interacting Genes/Proteins) (http://string-db.org/) to identify groups within them so as to prioritize genes for experimental validation. The analysis resulted in a network with high clustering coefficient of 0.58 and the average degree of ~13. The high clustering coefficient suggests that the network is also a community which may be involved in similar kind of function(s). The strategy resulted in four clusters (referred to as clusters A, B, C and D) with a maximum and minimum size of 16 and 3 genes respectively (Fig. 5). The cluster A has 9 members and was found to be enriched in genes controlling the cell communication process through signal transduction. The cluster B has 11 genes that were found to be related with processes like component organization and blood coagulation. The cluster C was a very small with only 3 genes. We could not find enriched processes for this cluster however, the genes of this cluster involve mainly in cell communication and cellular component organization and nicotine pharmacodynamics. The cluster D with 16 members was found to be enriched in the biological processes like cell differentiation and stress induced cell death.

Prior to validation through immunohistochemistry, the results of pathway analysis and the literature mining were carefully scrutinized in addition to clustering for the selection of the cluster representative. One representative from each cluster was selected (CALM3, ARRB1, FLNA and HTT from cluster A, B, C and D respectively) to represent diverse biological processes.

**Figure 6:**
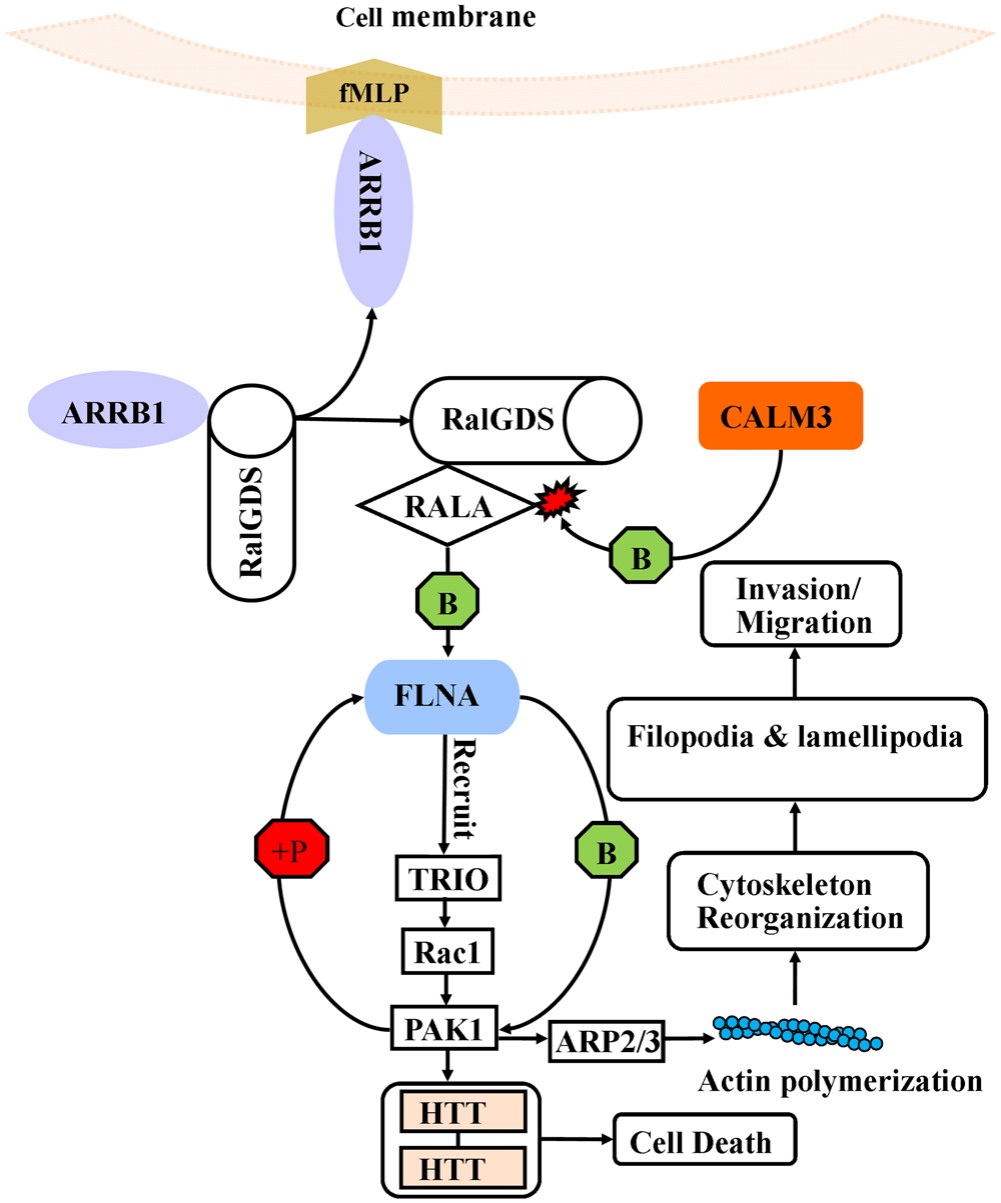
The proposed role of ARRB1, CALM3, FLNA and HTT in OSCC.

Thorough literature survey and pathway analysis led us to propose a mechanism (Fig. 6) to show the role of four genes ARRB1, CALM3, FLNA and HTT in OSCC. N-formyl-methionyl-leucyl-phenylalanine (fMLP) is a formylated peptide that disassociates ARRB1–Ral-GDS protein complexes and release Ral-GDS which results in activation of RALA and positively regulates actin cytoskeletal re-organization through FLNA^29^. Alternatively RALA is also involved in calcium/calmodulin-mediated intracellular signaling pathways where it is activated by Ca^2+^ via binding with CALM3^30^. FLNA is an effector protein of RALA which recruits a guanine nucleotide exchange factor (GEF) TRIO and catalyze the transition of Ras-like protein Rac1 from the GDP to the GTP bound form. Rac1-GTP then activates p21-activated kinase 1 (PAK1)^31,32^. FLNA also directly binds to PAK1 and gets phosphorylated. PAK1 promotes activation of actin polymerization by phosphorylating of Arp2/3 complex which ultimately helps in actin cytoskeletal reorganization^33^.

Actin cytoskeleton reorganization helps in development of protrusive actin structures called lamellipodia and filopodia which help in cell migration and invasion^34,35^. It has been reported that PAK1 bind to HTT and enhance the HTT-HTT interaction which leads to aggregation of HTT in the cytoplasm^36^. The HTT aggregation produces toxicity and causes the cell death^37^.

### Immunohistochemistry (IHC)

The expression of ARRB1, FLNA, CALM3 and HTT was found to be localized predominantly in the cytoplasm of the tumor cells.

#### ARRB1

**Figure 7:**
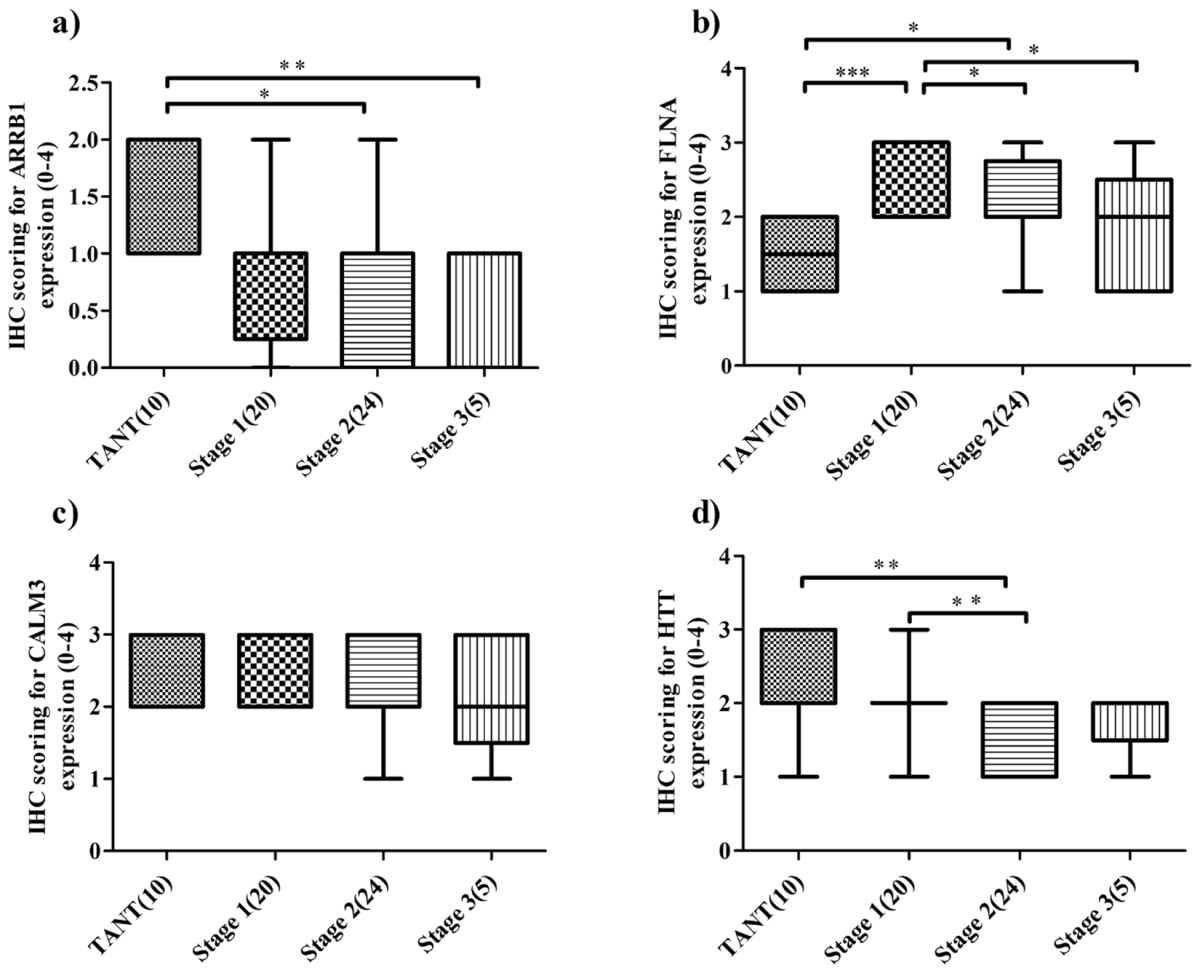
Box plot showing the stage wise expression of the genes on immunohistochemically stained TMA sections of OSCC samples. The scoring was done on scale of 0 to 4 [where 0: no staining, 1: 25% (mild staining), 2: 25–50% (medium staining), 3: 50–75% (moderate staining) and 4: ≥75% (strong staining)]. (**a**) ARRB1 expression was found to be significantly (p < 0.05) decreased from TANT (tumor adjacent normal tissue) to cancer but within the stages (stage 1 to stage 3) the expression did not show significant change. (**b**) Significantly (p < 0.05) upregulated expression of FLNA was found in the oral cancer patients as compared to the TANT samples but within the stages (stage 1 to stage 3) the expression goes down. (**c**) The expression of CALM3 did not show a significant change from TANT to cancerous samples or among the different stages of cancerous samples. (**d**) HTT showed a decreased expression as compared to the TANT samples. Especially, the expression significantly decreased from TANT to stage 2 and stage 1 to stage 2 (p < 0.05). The number of samples used is indicated in brackets.

**Figure 8:**
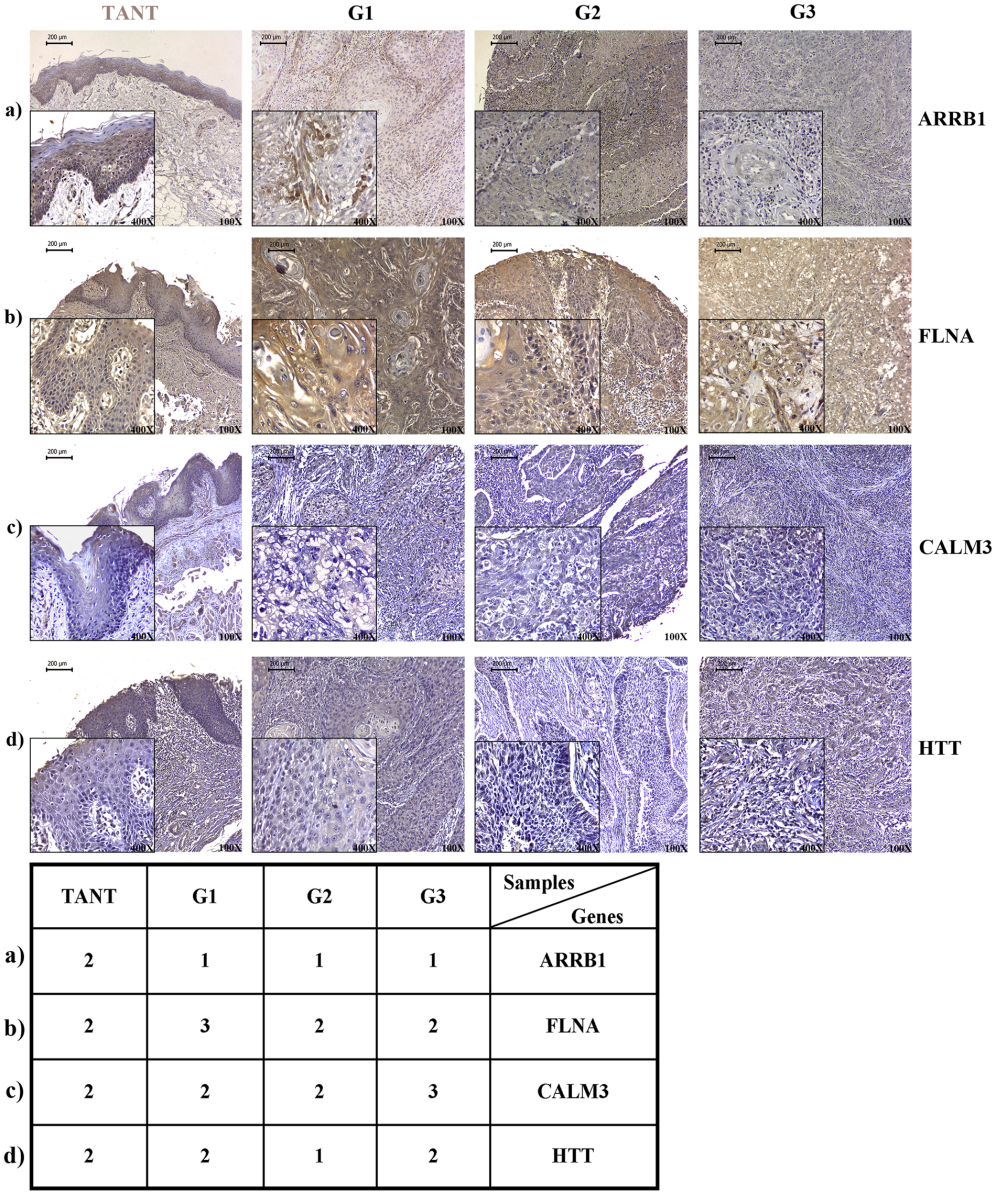
Representative figures of immunohistochemically stained TMA sections in TANT (tumor adjacent normal tissue), Grade 1(G1), Grade 2 (G2) and Grade 3 (G3) oral cancer patient samples. Upper panel: (**a**) Strong cytoplasmic staining of ARRB1 was observed in the TANT cells as compared to the cancerous cells. (**b**) FLNA showed a strong cytoplasmic staining in case of cancerous samples as compared to TANT samples (**c**) CALM3 showed cytoplasmic staining with no significant change between TANT to grade 1 but showed significant (p = 0.04) increases from grade1 to grade 3 cancer samples, (**d**) HTT showed a cytoplasmic staining which decreases from TANT to grade 3. All the images were captured using LEICA DM500 HD compound microscope at 100X magnification. The insert (400X) in the IHC images are the zoom in section of the same tissue section which provides a better visualization for the intensity of staining. Lower panel: table representing the corresponding score of each image in upper panel, which was calculated by taking the percentage of positive cells and intensity of staining.

In the current study, the mean expression index of ARRB1 was found to be 1.4, 0.95, 0.83 and 0.4 for TANT (Tumor Adjacent Normal Tissue), stage 1, stage 2 and stage 3 respectively. A statistical analysis by Mann-Whitney U test^38^ revealed the mean expression was significantly different between TANT and stage 2 (p = 0.043) and between TANT and stage 3 (p = 0.012) (Fig. 7a). The tissue expression was found to be decreased significantly between TANT and stage 2 (p = 0.039), between TANT and stage 3 (p = 0.007) by χ^2^ test in stage 2 and stage 3 as compared to TANT (Figs 7a and 8a). The grade wise comparison of its expression shows significant (p = 0.033) difference from TANT to grade 1 sample but not with the other grades (Supplementary Fig. S1a and Supplementary Table S7).

#### FLNA

The mean expression of FLNA was found to be 1.50, 2.55, 2.04 and 1.80 in TANT, stage 1, stage 2 and stage 3 respectively. The Mann-Whitney U test indicated that the difference in mean expression was statistically significant between TANT and stage 1 (p = 0.0002); TANT and stage 2 (p = 0.039); stage 1 and stage 2 (p = 0.0142) and stage 1 and stage 3 (p = 0.049). The χ^2^ test also revealed that the difference in expression, as compared to TANT, was significantly higher in stage 1 (p = 0.001) and stage 2 (p = 0.032) of OSCC (Fig. 7b and 8b). Among different stages, the FLNA expression was found to decrease progressively from stage 1 to stage 3 (p = 0.010 between stage 1 and stage 2; p = 0.018 between stage 1 and stage 3 by χ^2^ test (Fig. 7b). The grade wise association too, follows the same pattern (p < 0.05) (Fig. 8b, Supplementary Fig. S1b and Supplementary Table S7). Chi *et al*. also reported FLNA overexpression in OSCC as compared to TANT^39^. However, in their study they used only two samples that were inadequate to assess statistical significance.

#### CALM3

The mean expression index was found to be 2.30, 2.55, 2.29 and 2.20 for TANT, stage 1, stage 2 and stage 3 respectively. The stage-wise comparison did not show any significant (p-value > 0.05) difference in CALM3 expression between TANT and cancerous tissue (Fig. 7c). However, a grade-wise comparison showed a significant difference between grade 1 and grade 3 (p = 0.04) (Fig. 8c, Supplementary Fig. S1c and Supplementary Table S7).

#### HTT

The mean expression index of HTT was found to be 2.20, 2.50, 1.65 and 1.80 for TANT, stage 1, stage 2 and stage 3 respectively. The HTT was found to be significantly downregulated between TANT and stage 1 (p = 0.016), and between stage 1 to stage 2 (p = 0.002) by Mann-Whitney U test. The χ^2^ test also showed that the expression of HTT is significantly down-regulated in stage 2 as compared to TANT (p = 0.009), and in stage 2 as compared to stage 1 (p = 0.002) (Fig. 7d). In other stages, the difference in expression was not statistically significant (p > 0.05). A grade-wise comparison did not show any significant difference (Fig. 8d, Supplementary Fig. S1d and Supplementary Table S7).

## Discussion

Understanding the causative association between diseases and genes has been an important goal in computational biology. A highly successful strategy the so-called guilt-by-association (GBA) approach has been used widely to predict new candidate genes through their association with known genes. The following observations were made during the course of the study.

### The centrality measures can rank the genes in a meaningful way

Similar to social networks certain genes are more important in biological networks as they are involved in the control of essential processes. These genes are frequently found among disease genes. However, it is not easy to determine the relative importance of a protein in an exceedingly complex PPI network. The centralities rank network components according to their importance within the network structure. Thus, the network centralities help to determine significance of a protein in a PPI network.

To measure the relative importance of a protein in the HCGN, we calculated various centralities (total 11) dealing with the connectivity, distance, betweenness of a node, flow of information, contact with other important proteins etc. The ranks showed strong correlations with each other indicating a general agreement between different centralities. Many genes were found to have a high value of degree, betweenness, vulnerability etc. A majority of them were also connected with other important proteins as indicated by their high page rank value. The centrality values for four selected genes (ARRB1, CALM3, FLNA, HTT) were checked vis-a-vis with the average value of each centrality for all genes in the network. It can be seen from Supplementary Table S8 that the four selected genes have much better centrality scores as compared to the average.

Importantly, we wanted to evaluate the genes that are ranked higher in the network and also have more relevance to the diseases. To achieve this, we obtained information on the gene-disease associations from DisGeNET database^40^. The data relevant to all the genes in HCGN was extracted from “all gene disease associations” subset of DisGeNET. The genes, according to their consensus rank in HCGN, were pooled in ten equally sized bins i.e. the top ranked 10% genes were kept in the first bin followed by next 10% (i.e. genes ranked between top 10–20%) in the second bin and so on. The average number of diseases per gene in each bin was then calculated. Finally, the average numbers of diseases per genes were plotted against bin numbers to check if top ranked genes (that were placed in first or second bin) are actually related to higher number of diseases.

Interestingly, the results show that the genes that were ranked higher by the centralities are in fact related with more number of diseases as compared to the genes which were ranked lower as indicated by a high coefficient of determination (R^2^ = 0.72) (Supplementary Fig. S2). The results clearly indicate that the centrality measures can rank the genes in a meaningful way.

The biological relevance of the genes can be assessed through GO terms to which they are associated. The GO provides information for protein functions, processes, and localization. To identify new genes which are associated with the oral cancer related GO terms, we further identified enriched GO terms. The pruning of the identified GO terms further resulted in a set of more enriched terms. A majority of these GO terms were related to transport, vascular development, signaling or regulation. The results also showed enriched GO terms are related to the proteins which have associations with important cancer process such as regulation of apoptosis, vascular generation etc.

Finally, the enriched GO terms were combined with the top ranking genes in HCGN to predict new candidate genes. A list of total 39 candidate genes was obtained which were further analyzed.

### The combination of GO and network centralities is effective

It is always prudent to verify and validate the in-silico predictions. In the current case, verification was done by looking at differential gene expression in TANT vs. oral cancer samples. It is assumed that if a gene is differentially expressed in a disease then it may have a role in the disease process. However, it is not a necessary condition; a gene may also greatly influence the disease process even though its expression is not different significantly in TANT and disease states e.g. by means of epigenetic modifications or post-translational modifications etc. However being differentially expressed gives an important clue that the gene in question may be involved.

The analysis has shown that ~44% (17 out of 39) predicted genes were found to have differential mRNA expression in oral cancer tissues. There were twelve genes which were found to be upregulated while four genes were found to be downregulated. The gene ESR1 was found to be upregulated in the Expression Atlas while it was shown downregulated in Oncomine. A detailed literature survey further resulted in the identification of twenty-four genes which may be related to oral cancer in some way. Taken together the mRNA expression data and literature, twenty-nine out of the predicted thirty-nine genes have shown association with oral cancer. The results also cement our belief that combination of GO and network centralities is quite robust for the prediction of candidate genes. This led us to validate the remaining genes through experimental means.

### The cluster analysis identified four distinct clusters in the network

The cluster analysis resulted in the identification of four distinct clusters. It was interesting for us to see that these groups of genes are actually enriched in distinct processes like cell differentiation and stress-induced cell death, component organization and blood coagulation, signal transduction and nicotine pharmacodynamics.

### The immunohistochemistry corroborated the predictions

The ARRB1, also known as beta-arrestin1 is a ubiquitously expressed adaptor protein with a wide range of cellular and molecular functions and have shown to contribute to a number of diseases, including cancer^41–43^. The ARRB1 also induce hypoxia and may help in cancer cells growth and metastasis^44,45^. The IHC results clearly indicate that the ARRB1 expression gets downregulated with the advancing cancer stage.

The FilaminA (FLNA) is an actin-binding protein, known to be involved in cytoplasmic gelation, cell contraction, and spreading^46^. Cytoplasmic full-length FLNA promotes metastasis and migration through its actin-binding properties^47^. It acts as a scaffolding molecule and found to be overexpressed in multiple types of cancer, including prostate, breast, lung, hemangiomas, colon, melanoma, neuroblastoma, squamous cell carcinoma and hepatic cholangiocarcinoma^48–50^. Yue, *et al*. opined that the high level of FLNA in initial stage helps cancer cell detachment and migration from primary site, invasion to ECM (extra cellular matrix)^46^. Further the decreased expression of FLNA helps in re-establishment of the cancer cells to ECM at the secondary sites, thus helping in cancer metastasis^46^. In our study, the expression of FLNA showed a marked increase in the stage 1 patient samples as compared to the TANT. It then showed a downtrend as the cancer progresses to later stages i.e. stage 2 and 3. The same pattern was also seen in grade wise analysis for FLNA expression in the immunohistochemistry. These results indicate the proposed role of FLNA in cell invasion and metastasis.

The Calmodulin 3 (CALM3) is considered the major regulator of Ca^2+^ dependent signaling in all eukaryotes which assists in tumor growth, tumor-associated angiogenesis, metastasis and involved in various pathways associated with survival of the cancer cells^51^. The grade wise comparison showed no significant change in the CALM3 expression between TANT v/s Grades but from grade 1 to grade 3 the expression increases significantly. This indicates it may have a role in cancer progression.

The Huntingtin (HTT) is the protein mutated in Huntington’s disease (HD). Recently, it was reported that the expression of HTT is low at both mRNA and protein level in case of metastatic breast carcinomas. In the same report, it has been shown that downregulation of HTT was also associated with poor survival of breast cancer patients^52^. In our study also the HTT was found to be downregulated.

The detailed information about the genes used in the current study is given in Supplementary Table S9. The columns indicate the status of a gene in different datasets (HIPPIE, TCGA, Oral cancer databases, HCGN, set A/B/C).

## Conclusion

Though our work has been inspired by many other *in-silico* studies, there is no universally accepted protocol for the prediction of potential candidate genes. In this approach we have used an integrated approach where each of the component was optimized separately e.g. the cut-off for top ranking genes was done using a consensus ranking, further the ranking was checked for robustness by validating through DisGeNET database. The selection of enriched GO terms was also optimized using a variety of combinations and finally a trimming was done using REVIGO. In this way we were able to predicted 39 potential candidate genes.

Initially, an investigation of the predictions was done using literature, Oncomine (mRNA) and Expression ATLAS (mRNA and RNA-seq). The investigation showed that out of 39 genes, we have 29 positive predictions which indicate the robustness of the method. The experimental validation of predicted genes indicated the robustness of the current approach. The clustering, pathway analysis and literature survey led to the generation of a hypothesis for the role of four genes (ARRB1, FLNA, CALM3 and HTT) in OSCC. Further experimental validation by immunohistochemistry indicates that these genes are related to oral cancer. It is hoped that the identified genes will aid future drug discovery efforts.

## Material and Methods

In this study we have tried to identify the candidate genes involved in OSCC using the below integrated approach of PPI network analysis and GO analysis. The flow chart of the detailed methodology is shown in Fig. 1.

### Data collection

The cancer genes were obtained from TCGA-generanker (http://cbio.mskcc.org/tcga-generanker/sources.jsp, accessed on 21^st^ April 2014). The generanker has been utilized in many such studies^53,54^. The TCGA contains a total of 7658 cancer candidate genes derived from 39 gene lists; each list has been given a score of 0.25 to 1; where 1 is high confidence value of the cancer genes. In the current study, we have selected 5873 candidate genes with a cutoff score ≥ 1.

The PPIs can be detected through different experimental approaches and have been collected in several expert-curated databases. We have used HIPPIE version 1.7 (2014) database for the present work^20^. This database is generated by pooling information from many other PPI databases e.g. BioGrid, DIP, HPRD, MINT, BIND, and MIPS, etc. Collectively, it contained 15519 proteins and 193486 interactions at the time of the current work. HIPPIE’s scoring scheme has been optimized to reflect the amount and quality of evidence for a given PPI. This score has been calculated from three major criteria i.e. number of studies in which an interaction was detected, number and quality of experimental techniques used to measure an interaction, and number of non-human organisms in which an interaction was reproduced. This database contains only those interactions that have been experimentally identified in human proteins. Further, different scores (0–10) have been given to various experiment types e.g. methods that can ascertain interactions with the highest reliability (X-ray crystallography) are assigned the highest scores, complementation-based assays, and affinity-based technologies are scored with an average value of 5, and co-localization or co-sedimentation based methods are scored lowest. The overall score is calculated in such a way that it gives scores between 0 (no confidence) to 1 (high confidence) for a given PPI. In the current study, we have used HIPPIE score cutoff of 0.63 (corresponding to the second quartile of the HIPPIE score distribution) to select more reliable interactions (148707) among 14391 proteins in the current analysis.

*Homo sapiens* related GO terms for biological process, molecular function, and cellular component were extracted from GO database (www.geneontology.org, version July 2015).

The genes related to oral cancer were acquired from OrCGDBv.2 (2011) and HNOCDB v1(2012). The OrCGDB contains total 374 genes, and the HNOCDB contains 133 oral cancer genes. The HNOCDB contains information about genetic mutations, methylations, and polymorphism, etc. and expression profiles. It also contains a chromosomal map of head-neck and oral cancer genes and experimental validation for their inclusion in HNOCDB. These two databases were combined, and 465 oral cancer genes were obtained. These genes were subsequently used in this study as known oral cancer genes. All the data reported above was collected during the course of this work.

### Construction of human cancer gene network

The interactions between cancer genes were extracted from HIPPIE database and a network was constructed. The orphan nodes self-loop, and parallel edges were removed. The fully connected remaining sub-network of PPI was defined as human cancer gene network (HCGN) for the current study. Genes, which were common among oral cancer (465) and HCGN (4704) genes, were defined as oral cancer genes in HCGN (Set B 297).

### Topological analysis of HCGN

The HCGN was analyzed for type of graph and the graph properties. The topological parameters like average degree, degree distribution, graph diameter, average distance, graph density, modularity, global efficiency and average clustering coefficient were calculated to characterize the interaction network. The degree distribution is the probability that a randomly chosen node have the K number of connection. The degree distribution graph is used to investigate the network structure type where the degree is represented by X-axis and degree distribution on the Y-axis respectively. The graph diameter tells the maximum numbers of nodes needed to be traveled to reach from one node to another node in the network. In other terms, it can indicate the ease with which the proteins can interact and affect their reciprocal functions. Average degree signifies the average number of connection per node. The graph density indicates the status of completion of the graph, as maximum density for the complete graph is 1. Modularity is a measure of the strength of division of network in different modules. Average clustering coefficient tells the probability of each node to cluster with other nodes. The graph parameters which are called network centralities were also calculated. The centrality measure of each node indicates its importance in the network. Centralities, for a node, are usually based on the number of its connections (e.g. degree) and shortest path distance (e.g. betweenness) with other nodes. Each centrality gives the significance of a node in a different way for the network under consideration. In the current study eleven different centralities viz. degree, closeness, radiality, shortest-path betweenness, current-flow betweenness, current-flow closeness, centroid, page rank, vulnerability, stress and eigenvector were calculated using software Centibin, Cytoscape 3.2.1, and in-house code^55,56^. Detailed information about these centralities is given in the supplementary data.

The genes were ranked according to their importance in the network based on the calculated centrality parameters. Initially the ranking was done using individual centrality i.e. each gene was ranked by each centrality. These ranks were then used to create a consensus ranking (rank-by-rank consensus scoring) and the genes were ranked based on the consensus score. This kind of scoring makes it easy to combine the ranks by two or more centralities without the need of normalization. This also avoids bias because of a single centrality. Such rank-by-rank consensus scoring has been successfully used in other areas like screening of chemical databases to identify candidate molecules. In the current study, as shown later, this scheme was found to perform better than other ranking schemes.

The rank correlation analysis was performed to check inter-correlation among centralities. This analysis is important to prevent specific bias that may get introduced in consensus score because of very high inter-correlation between two or more centralities.

The rank correlation between two centralities was calculated by spearman’s rank correlation coefficient *ρ*

$$\rho =1-\frac{6\sum {d}_{i}^{2}}{n({n}^{2}-1)}$$

where *d* is the difference of the rank given by two different centralities for one gene and *n* is the total number of nodes or genes. The coefficient *ρ* can range from −1 to 1 where 1 stands for perfectly positive correlation zero (0) for no correlation and −1 perfectly negative correlation.

The idea to rank the genes by different centralities was to identify important nodes in the network that may have control over the other nodes in the network. These hubs or information critical nodes are known to be associated with many other cancers. Therefore, the presence of oral cancer genes in HCGN (Set B) in top 2%, 5%, 7% and 10% of ranked gene list by different centralities was checked to assess the association of these hubs with oral cancer. Additionally, consensus rankings by taking different centralities were also assessed to identify the best combination of centralities for prediction of oral cancer genes.

It was found that a combination of top 5% genes from 10 different centralities was having a significant number of genes known to be associated with oral cancer (details shown in results). This observation supported our assumption that the nodes with high importance may be associated with oral cancer by virtue of their control over flow of information in the network.

### GO enrichment analysis

GORILLA was used for the identification of enriched GO terms. It is a web-based application that identifies enriched GO terms in a target list of genes compared to a background list of genes. It can also identify enriched GO terms, in lists with ranked genes, without the need of explicit target and background sets. GORILLA employs a flexible threshold statistical approaches to discover GO terms that are significantly enriched as compared to background list. GORILLA’s unique features and advantages over other threshold free enrichment tools include rigorous statistics, fast running time and intuitive graphical representation.

The GO enrichment analysis for oral cancer genes was performed using GORILLA online tool in two list mode with p-value < 0.001. The target was oral cancer genes (total 465) and the background was cancer genes (total 6023). The total of 6023 genes contain 5872 cancer candidate genes (5873 from TCGA list-one gene could not be mapped to gene id) and 151 (out of total 465) oral cancer genes which is not listed in TCGA. The target and background were used to extract all the enriched GO terms specific to oral cancer.

GO term enrichment analysis by the majority of the tools generally gives p-value and enrichment score. The p-value gives the significant level while the enrichment score gives the over/under representation of a particular term in a given gene list. However, it has been reported that highly explored GO terms are more significantly enriched as compared to less explored GO terms. An inspection of the enriched GO terms revealed that there are many terms having significant overlap with other enriched GO terms; additionally, there are many terms that were too general e.g. metabolic process (GO:0008152). Therefore, it was necessary to prune the identified terms to reduce redundancy and to make the information obtained from GO more interpretable.

There are many tools like AmiGO, Ontolizer and RedundancyMiner that usually cluster the GO terms to the higher level or to more general term and help reduce redundancy. In the current study, we have used REVIGO which implements a clustering procedure conceptually similar to the hierarchical clustering methods such as the neighbor-joining approach. It selects a representative GO term out of many similar or overlapping terms which reduce functional redundancies using semantic similarity measures. The semantic similarity measures between GO terms rely on pre-computed information content (IC) for the GO terms. The IC is calculated as a negative logarithm of the GO term’s relative frequency in a reference database. The REVIGO online server with default settings was used to remove the redundant GO terms from the enriched GO terms. The remaining GO terms were then used to select associated genes in HCGN.

### Prediction of candidate genes for oral cancer

Having identified the enriched GO terms and important hubs in the network, the next task was to utilize the obtained information to predict the candidate genes for oral cancer. To this end, the top ranking 5% genes from consensus ranking list (defined as Set A), oral cancer genes in HCGN (defined as Set B) and genes related to disease associated enriched GO terms (defined as Set C) were analyzed. A gene was predicted to be a candidate oral cancer associated gene if it meets all of the following three criteria:

1. It should be highly ranked by centralities (member of Set A).
2. It should be associated with enriched GO terms (member of Set C).
3. It should not be hitherto known for association with oral cancer (not a member of Set B).

Thus a gene common between Set A and Set C but not present in Set B was defined as candidate oral cancer gene (Fig. 1).

### Pathway and Functional analysis

The pathway analysis was performed on the predicted potential candidate genes. A pathway is considered to be enriched in a gene list based on an ‘expected’ value. It is calculated by the fraction of the genes in a list related to the pathway (observed value) compared with fraction of the genes in a pathway to that of the whole gene database (expected value). If the observed value is greater than expected value, the pathway is considered enriched^24^.

The DAVID version 6.7 was used for the functional enrichment analysis in the current studies^27^. The DAVID has various tools for gene annotation, GO enrichment analysis, PPI analysis, pathway analysis and visualization etc. It was used to identify significantly enriched (p-value < 0.01) functions amongst a set of predicted candidate genes.

Additionally, The PANTHER classification system designed for the classification of proteins/genes to facilitate high-throughput analysis was used for the pathway enrichment analysis for predicted candidate genes using a p-value cutoff of 0.01^24^.

### Evidence in literature/databases for predicted candidate genes

#### Oncomine database

The Oncomine is a cancer microarray database and web-based data-mining platform aimed at facilitating discovery from genome-wide expression analyses. It can give information on differential gene expression in various types of cancer. The pathological and clinical data for genes is also available for better understanding of different stages of cancer.

#### Expression Atlas

It provides information on gene expression patterns under different biological conditions. It contains both the mRNA and RNA-seq data related with gene expression.

In the current study we have used these databases to identify differentially expressed genes in oral cancer with the assumption that if a gene is differentially expressed in oral cancer, it may have an association with oral cancer.

### STRING database

The STRING v10 is a database which provides the interactions of proteins by integrating information from high-throughput experimental data, from the mining of databases and literature, and from predictions based on genomic context analysis. It also contains information about protein domains and structures. It gives an association score for two interacting proteins, calculated using various parameters like neighborhood score, fusion, co-occurrence, homology, co-expression, experimental, database and text mining score etc. A high score indicates greater association confidence. String also provide tools to analyze the network in many ways e.g. enrichment of GO, pathways, and clustering etc. In the current study, the k-means clustering as implied in STRING was used to cluster the genes^28^. The clustering was done on the basis of evidence score and connection cutoff was kept at 0.40.

### Immunohistochemistry (IHC)

Four human Tissue microarray slides (TMA) were procured from US Biomax (http://www.biomax.us/tissue-arrays/Oral_Cavity/). One slide/gene was used for the immunohistochemical analysis. Each slide includes 50 samples of OSCC and 10 TANT. The stage wise information for 50 OSCC samples were distributed as: stage 1 = 20, stage 2 = 24 and stage 3 = 5 tissues samples. We did not considered 1 tissue spot (A2) which was of stage 4a. The grade wise information for 50 OSCC samples were distributed as: grade 1 = 38, grade 2 = 6 and grade 3 = 5 tissue samples. The E5 spot was considered in both grade 1 as well as in grade 2 as per the data sheet provided, where the grade information was not provided for 2 samples. Standard immunohistochemical staining procedure was performed to check the expression of the selected genes with slight modifications as described earlier^57^. The slides were deparaffinized and processed through down-gradation of ethanol (from 100% to 70%). Then the slides were hydrated with deionized water, and antigen unmasking was done by boiling with antigen unmasking solution (Cat No. H-3301, Vector lab) in pressurized condition. Slides were blocked for 1hr at room temperature with blocking solution and antibody blocking was done overnight at 4 °C in a moist chamber. Then VECTASTAIN Elite ABC Kit (cat No. PK-7200, Vector lab) was used for processing as per the user’s manual provided and the slides were developed by DAB Peroxidase Substrate Kit (Cat No. SK-4100, Vector lab). The TMA slides were blocked with respective antibodies (ARRB1, FLNA, CALM3 and HTT) and IHC staining of TMA tissue spots was scored by inter-observers (histopathologist) manually to avoid bias. The score from 0 to 4 was given on the basis of percentage of positive cells and intensity of staining as follows: 0%, no staining = 0; 25%, mild staining = 1; 25–50%, medium staining = 2; 50–75%, moderate staining = 3 and ≥ 75%, strong staining = 4) had been described in earlier studies^58^. All sections were scored in a semi-quantitative manner according to the method described previously, which reflects both the intensity and percentage of cells staining. The corresponding score for each sample in each TMA slide for the four genes was calculated separately.

### Antibodies used

Arrestin beta 1 Antibody (ARRB1), was obtained from Novus biochemical (Cat No. NB110-55485). FILAMIN A (FLNA) and Anti–Huntingtin Protein (HTT) a.a. 181–810, clone 1 HU–4C8 were obtained from Millipore (Cat No. MAB1680 and MAB2166 respectively). CaM (FL-149) was obtained from Santa Cruz biotechnology (Cat No. SC-5537).

### Statistical Analysis

Statistical analysis for the immunohistochemistry data was performed using GrapPad Prism 5 software. The categorical data was compared for difference using the χ^2^ test. Fisher’s exact test was used to detect association of a gene with oral cancer. A nonparametric unpaired Mann-Whitney test^38^ was used to compare the mean of two independent groups e.g. normal and cancer’s stage and grade. The p-value < 0.05 was considered significant for each statistical test^59,60^.

## Electronic supplementary material

Supplementry data

Supplementary Table S9
