# Author Correction: Identification of oral cancer related candidate genes by integrating protein-protein interactions, gene ontology, pathway analysis and immunohistochemistry

**DOI:** 10.1038/s41598-018-23236-2

**Published:** 2018-04-19

**Authors:** Ravindra Kumar, Sabindra K. Samal, Samapika Routray, Rupesh Dash, Anshuman Dixit

**Affiliations:** 1 Institute of Life Sciences, Nalco Square, Bhubaneswar, 751023 Odisha India; 2 Manipal University, Manipal, 576104 Karnataka India; 3 Siksha ‘O’ Anusandhan University, Bhubaneswar, 751003 Odisha India; 4 All India Institute of Medical Sciences, Sijhua, Bhubaneswar, 751019 Odisha India

Correction to: *Scientific Reports* 10.1038/s41598-017-02522-5, published online 30 May 2017

This Article contains errors.

In Table 1, incorrect values for ‘Consensus Ranking’ are given. The correct Table 1 appears below as Table 1.

**Table 1:** The distribution of oral cancer genes in HCGN in top ranked lists.

	Centrality	Degree	Closeness	Centroid	SPB	Eigen vector	Page rank	CFC	CFB	Stress	Vulnerability	Unique pooled genes	Consensus Ranking
**Top 2%**	Oral cancer genes^#^	2	22	18	24	26	13	27	21	23	21	37	30
	Total genes	4	67	46	76	90	47	90	71	74	84	152	91
	Precision*	50.00	32.84	39.13	31.58	28.89	27.66	30.00	29.58	31.08	25.00	24.34	30.77
**Top 5%**	Oral cancer genes^#^	5	41	31	43	46	32	51	43	44	45	71	56
	Total genes	9	170	109	193	221	121	221	181	180	211	359	227
	Precision*	55.56	24.12	28.44	22.28	20.81	26.45	23.08	23.76	24.44	21.33	19.77	24.67
**Top 7%**	Oral cancer genes^#^	7	55	37	59	68	42	65	56	56	58	94	68
	Total genes	13	237	157	269	309	168	308	252	251	295	517	313
	Precision*	61.54	23.21	23.57	21.93	22.01	25.00	21.10	22.22	22.31	19.66	18.18	23.85
**Top 10%**	Oral cancer genes^#^	10	75	52	77	85	53	83	71	73	70	118	88
	Total genes	18	354	230	383	442	239	440	359	357	423	762	443
	Precision*	44.44	21.19	22.61	20.10	19.23	22.18	18.86	19.78	20.45	16.55	15.48	19.87

^*^Precision is the percentage of oral cancer genes in total genes. ^#^Common out of 297 oral cancer genes in HCGN.

As a result,

“The precision score for top 2%, 5%, 7%, and 10% of the consensus ranked genes was found to be 30.77, 24.67, 23.85 and 19.87 respectively (Table 1). This indicates that consensus ranking is clearly better than individual centralities or unique pooled genes (Table 1). The precision decreases as we move from top ranked 2% to top ranked 10% genes. Though the top ranked 2% genes comparatively showed greater fraction of oral cancer genes in HCGN, the total number of unique oral cancer genes was very less (28 out of total 91 genes, Table 1).”

should read:

“The precision score for top 2%, 5%, 7%, and 10% of the consensus ranked genes was found to be 32.97, 24.67, 21.73 and 19.86 respectively (Table 1). This indicates that consensus ranking is clearly better than individual centralities or unique pooled genes (Table 1). The precision decreases as we move from top ranked 2% to top ranked 10% genes. Though the top ranked 2% genes comparatively showed greater fraction of oral cancer genes in HCGN, the total number of unique oral cancer genes was very less (30 out of total 91 genes, Table 1).”

In Table 3, incorrect values for ‘HCGN Ranking’ and ‘Degree with known oral cancer genes’ are given. The correct Table 3 appears below as Table 2.

**Table 2:** The candidate oral cancer genes.

S. No.	Gene ID	Gene Symbol	HCGN Ranking	Degree with known oral cancer genes	Expression Atlas (mRNA)	Oncomine mRNA expression^*^
**1**	3320	HSP90AA1	1	55		Up
**2**	8452	CUL3	2	41		NA
**3**	2099	ESR1	3	59	Up	Down
**4**	3312	HSPA8	4	30	Up	Up
**5**	1499	CTNNB1	5	32		NA
**6**	6714	SRC	6	32		NA
**7**	4088	SMAD3	7	20	Up	NA
**8**	5071	PARK2	8	21	Down	Down
**9**	5970	RELA	9	23		NA
**10**	207	AKT1	10	28		NA
**11**	408	ARRB1	11	18		NA
**12**	7186	TRAF2	12	21		NA
**13**	1457	CSNK2A1	13	25		Up
**14**	7013	TERF1	14	15		Up
**15**	5591	PRKDC	15	25		Up
**16**	3064	HTT	15	15		NA
**17**	23411	SIRT1	17	20		NA
**18**	5518	PPP2R1A	18	14		NA
**19**	10014	HDAC5	19	21		NA
**20**	4176	MCM7	20	21		Up
**21**	3676	ITGA4	21	38		Up
**22**	1432	MAPK14	22	15		NA
**23**	808	CALM3	23	12		NA
**24**	805	CALM2	23	12		NA
**25**	801	CALM1	23	12		NA
**26**	4851	NOTCH1	26	10		NA
**27**	5515	PPP2CA	27	16		Up
**28**	2316	FLNA	28	14	Up	Up
**29**	5894	RAF1	29	19		NA
**30**	8841	HDAC3	30	12		NA
**31**	8826	IQGAP1	31	11	Down	NA
**32**	5747	PTK2	32	23		Up
**33**	6657	SOX2	33	7	Down	NA
**34**	6195	RPS6KA1	34	9		NA
**35**	5580	PRKCD	35	15		NA
**36**	3717	JAK2	36	15		NA
**37**	998	CDC42	37	5		Up
**38**	6709	SPTAN1	38	10		NA
**39**	3688	ITGB1	39	14	Down	NA

^*^The mRNA expression status (oral cancer vs normal) of a gene as reported in the Oncomine database and expression ATLAS. NA = Not reported.

