# Publisher Correction: Identification of oral cancer related candidate genes by integrating protein-protein interactions, gene ontology, pathway analysis and immunohistochemistry

**DOI:** 10.1038/s41598-018-26708-7

**Published:** 2018-05-24

**Authors:** Ravindra Kumar, Sabindra K. Samal, Samapika Routray, Rupesh Dash, Anshuman Dixit

**Affiliations:** 1 Institute of Life Sciences, Nalco Square, Bhubaneswar, 751023 Odisha India; 2 Manipal University, Manipal, 576104 Karnataka India; 3 Siksha ‘O’ Anusandhan University, Bhubaneswar, 751003 Odisha India; 4 All India Institute of Medical Sciences, Sijhua, Bhubaneswar, 751019 Odisha India

Correction to: *Scientific Reports* 10.1038/s41598-017-02522-5, published online 30 May 2017

This Article and the Author Correction published on 19th April 2018 contain errors in Table 1, where incorrect values for ‘Consensus Ranking’ are given. The correct Table 1 appears below.

**Table 1:** The distribution of oral cancer genes in HCGN in top ranked lists.

	Centrality	Degree	Closeness	Centroid	SPB	Eigen vector	Page rank	CFC	CFB	Stress	Vulnerability	Unique pooled genes	Consensus Ranking
**Top 2 %**	Oral cancer genes^#^	2	22	18	24	26	13	27	21	23	21	37	30
	Total genes	4	67	46	76	90	47	90	71	74	84	152	91
	Precision*	50.00	32.84	39.13	31.58	28.89	27.66	30.00	29.58	31.08	25.00	24.34	32.97
**Top 5 %**	Oral cancer genes^#^	5	41	31	43	46	32	51	43	44	45	71	56
	Total genes	9	170	109	193	221	121	221	181	180	211	359	227
	Precision*	55.56	24.12	28.44	22.28	20.81	26.45	23.08	23.76	24.44	21.33	19.77	24.67
**Top 7 %**	Oral cancer genes^#^	8	55	37	59	68	42	65	56	56	58	94	68
	Total genes	13	237	157	269	309	168	308	252	251	295	517	313
	Precision*	61.54	23.21	23.57	21.93	22.01	25.00	21.10	22.22	22.31	19.66	18.18	21.73
**Top 10 %**	Oral cancer genes^#^	8	75	52	77	85	53	83	71	73	70	118	88
	Total genes	18	354	230	383	442	239	440	359	357	423	762	443
	Precision*	44.44	21.19	22.61	20.10	19.23	22.18	18.86	19.78	20.45	16.55	15.48	19.86

*Precision is the percentage of oral cancer genes in total genes. ^#^Common out of 297 oral cancer genes in HCGN.

