# Supplementary material for: Identification of oral cancer related candidate genes by integrating protein-protein interactions, gene ontology, pathway analysis and immunohistochemistry
Source: Sci Rep. 2017 May 30;7:2472. doi: 10.1038/s41598-017-02522-5 (PMC5449392; doi:10.1038/s41598-017-02522-5)
Supplement: Supplementary file 1 — Supplementry data [file 41598_2017_2522_MOESM1_ESM.doc]

**Identification of oral cancer related candidate genes by integrating protein-protein interactions, gene ontology, pathway analysis and immunohistochemistry**

Ravindra Kumar^1^, Sabindra K. Samal^1, 2^, Samapika Routray ^3, 4^, Rupesh Dash^1^, Anshuman Dixit^1*^

^1^Institute of Life Sciences Nalco Square Bhubaneswar 751023 Odisha India

^2^Manipal University, Manipal, 576104, Karnataka, India

^3^Siksha ‘O’ Anusandhan University, Bhubaneswar 751003 Odisha India

^4^All India Institute of Medical Sciences, Sijhua, Bhubaneswar, 751019, Odisha, India

**corresponding author:* [*anshumandixit@gmail.com*](mailto:anshumandixit@gmail.com)

**Definition of eleven centralities used for the study**

Let G is a directed or undirected graph which is represented as G = (V, E) where V is a set of vertexes or nodes and E is the set of edges or interactions. Total number of nodes is n in the network and deg(v) denotes the degree of the vertex v in an undirected graph. dist(v, w) denotes the length of a shortest path between the vertices s and t; σ_st_ denotes the number of shortest paths from s to t and σ_st_ (v) the number of shortest path from s to t that use the vertex v. Let A be the adjacency matrix of the graph G [^1-6^](#_ENREF_1).

**Degree:** The degree is defined by the total number of direct connections of a node to other nodes in the network. It indicates the relative importance or role of a node in a network e.g. in a PPI network a node with high degree (connections) might indicate a central regulatory role.

C_deg_ (v) = deg(v) ….(1)
For directed graph in and out degree is considered

**Closeness:** It is the reciprocal of the sum of shortest paths between a node and all other nodes in the graph. If the sum of distances is smaller it will result in a higher value meaning the node on average is in closer proximity to other nodes. It can indicate how long it will take to spread information from a node to all other nodes sequentially. However, high and low values are more meaningful when compared to the average closeness of the network. The closeness of a node in a protein-signaling network can be inferred as the probability of a protein to be functionally relevant for other nearby proteins.

C_clo_(v)
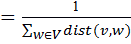
 ….(2)
**Radiality:** It gives an indication of a node's proximity or distance with respect to other nodes. A high value of the radiality means that, with respect to the diameter, the node is generally closer to the other nodes. On the other hand, a low radiality indicates that the node is peripheral. A protein with high radiality will be easily central to the regulation of other proteins while out of touch i.e. irrelevant for others.

C_rad_ (v)
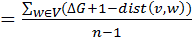
 ….(3)
**Shortest-path betweenness:** It represents the contribution of a node v, towards communication between all nodes pairs.

C_spb_ (v) =
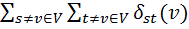

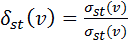
 ….(4)
**Current flow betweenness:** A common criticism for shortest-path based measures is that they do not take into account spread along non-shortest paths. The current flow betweenness tries to address this problem and appropriately it is also called the random-walk betweenness. The current flow betweenness of a node v is the average of the current flow over all source–target pairs

C_cfb_(v)
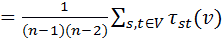
 ….(5)
Where
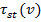
 equals the fraction of electrical current running over vertex v in an electrical network

**Current flow closeness:** Current-flow closeness is a variant of shortest-path closeness centrality for an alternative model of information spreading. It uses the notion of resistance distance. The resistance distance between two nodes is short if there are many short paths connecting the two nodes.

C_cfc_(v)
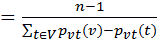
 ….(6)
Where
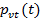
 equals the potential difference in an electrical network.

**Centroid:** The centroid value is the most complex node centrality index and is computed by focusing the calculus on couples of nodes (v, w) and systematically counting the nodes that are closer (in terms of the shortest path) to v or to w. A node v with the highest centroid value is the node with the highest number of neighbors separated by the shortest path to v,

C_cen_ (v) = min {f(v, w):
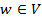
\{v}} ….(7)
Where f (v,w) =
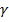
_v_(w) -
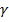
_w_(v) and
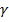
_v_(w) denotes the number of vertices that are closer to v than to w.

C_rad_(v)
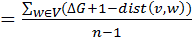
 ….(8)
**Page rank:** The page rank is an algorithm for ranking of website pages by search engine Google. A node is important, if it linked from many other important and frugal nodes. There are three distinct factors that determine the PageRank of a node: (i) the degree (ii) the frugality of the linkers, and (iii) the centrality of the linkers.

C_pr_ =dP C_pr_ + (l - d)
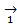
 ….(9)
Where P is the transition matrix and d is the damping factor.

**Eigen vector:** Scores the relative importance of all nodes in the network by weighting connections to highly important nodes more than connections to nodes of low importance. It can be viewed as a kind of weighted degree, where both the number and score of the neighbors are important. A high value indicates that the node is being visited a lot and is interacting with several important proteins. It may be indicative of a central regulatory role.

λC_eiv_=AC_eiv_ ….(10)
The dominant eigenvalue of A is used as eigenvector.

**Stress:** It is calculated by measuring the number of shortest paths passing through a node. It can indicate the relevance of a protein as functionally capable of holding together communicating nodes. The higher is the value higher will be the relevance of the protein in connecting regulatory molecules. However, high stress does not necessarily mean that the node is critical to communication between nodes whose paths are passing through it.

C_spb_ (v) =
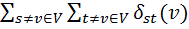

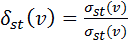
 ….(11)
**Vulnerability:** This is an important parameter that indicates the change in efficiency when a particular node is removed from the network. The efficiency of the network is calculated from the inverse sum of the shortest path of between any two nodes [^7^](#_ENREF_7).

The efficiency of the network is defined as

E(G)
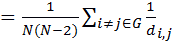
 ….(12)
Where G is the graph, d_i,j_ is the shortest path between node i and j and N(N-1) is the normalization constant. The vulnerability of the node v is calculated from

C_v_(v)= E(G) – E(G-v_i_) ….(13)
E(G-v_i_) is the efficiency of the network after removing the node v.

**Supplementary Table S1: Unique ranks given by each centrality.**

| Centrality | Degree | Closeness | Radiality | Centroid | SPB | Eigen vector | Page Rank | CFB | CFC | Vulnerability | Stress |
| --- | --- | --- | --- | --- | --- | --- | --- | --- | --- | --- | --- |
| Unique scores | 181 | 3131 | 3131 | 2039 | 3797 | 4368 | 2364 | 3552 | 4359 | 4219 | 3541 |

**Supplementary Table S2: Rank correlation of different centralities.**

|  | Degree | Closeness | Radiality | Centroid | SPB | Eigenvector | Page rank | CFB | CFC | Stress | Vulnerability |
| --- | --- | --- | --- | --- | --- | --- | --- | --- | --- | --- | --- |
| Degree | 1.00 | 0.87 | 0.87 | 0.95 | 0.91 | 0.90 | 0.98 | 0.97 | 0.99 | 0.92 | 0.85 |
| Closeness | 0.87 | 1.00 | 1.00 | 0.93 | 0.79 | 0.98 | 0.82 | 0.80 | 0.90 | 0.83 | 0.91 |
| Radiality | 0.87 | 1.00 | 1.00 | 0.93 | 0.79 | 0.98 | 0.82 | 0.80 | 0.90 | 0.83 | 0.91 |
| Centroid | 0.95 | 0.93 | 0.93 | 1.00 | 0.84 | 0.95 | 0.90 | 0.88 | 0.96 | 0.88 | 0.86 |
| SPB | 0.91 | 0.79 | 0.79 | 0.84 | 1.00 | 0.79 | 0.94 | 0.97 | 0.88 | 0.99 | 0.88 |
| Eigenvector | 0.90 | 0.98 | 0.98 | 0.95 | 0.79 | 1.00 | 0.84 | 0.82 | 0.92 | 0.83 | 0.90 |
| Page rank | 0.98 | 0.82 | 0.82 | 0.90 | 0.94 | 0.84 | 1.00 | 0.99 | 0.96 | 0.95 | 0.85 |
| CFB | 0.97 | 0.80 | 0.80 | 0.88 | 0.97 | 0.82 | 0.99 | 1.00 | 0.95 | 0.96 | 0.85 |
| CFC | 0.99 | 0.90 | 0.90 | 0.96 | 0.88 | 0.92 | 0.96 | 0.95 | 1.00 | 0.91 | 0.85 |
| Stress | 0.92 | 0.83 | 0.83 | 0.88 | 0.99 | 0.83 | 0.95 | 0.96 | 0.91 | 1.00 | 0.91 |
| Vulnerability | 0.85 | 0.91 | 0.91 | 0.86 | 0.88 | 0.90 | 0.85 | 0.85 | 0.85 | 0.91 | 1.00 |

Supplementary Table S3: The ranking of genes (top 10) by different centralities.

| **Gene ID** | **Gene Symbol** | **Degree** | **Closeness** | **Centroid** | **SPB** | **Eigen**  **vector** | **Page**  **rank** | **CFB** | **CFC** | **Stress** | **Vulnerability** | **Sum of**  **ranks** | **Consensus**  **Rank** |
| --- | --- | --- | --- | --- | --- | --- | --- | --- | --- | --- | --- | --- | --- |
| 1994 | ELAVL1 | 1 | 1 | 1 | 1 | 6 | 1 | 1 | 1 | 1 | 1 | 15 | 1 |
| 3320 | HSP90AA1 | 2 | 3 | 2 | 2 | 2 | 2 | 2 | 2 | 2 | 4 | 23 | 2 |
| 7157 | TP53 | 3 | 2 | 3 | 4 | 1 | 5 | 6 | 3 | 6 | 8 | 41 | 3 |
| 4609 | MYC | 5 | 6 | 7 | 3 | 7 | 3 | 3 | 6 | 3 | 2 | 45 | 4 |
| 8452 | CUL3 | 4 | 5 | 6 | 5 | 5 | 4 | 4 | 4 | 4 | 5 | 46 | 5 |
| 2099 | ESR1 | 6 | 4 | 4 | 8 | 3 | 6 | 10 | 5 | 7 | 7 | 60 | 6 |
| 4193 | MDM2 | 7 | 14 | 8 | 10 | 4 | 7 | 8 | 7 | 8 | 12 | 85 | 7 |
| 2885 | GRB2 | 8 | 9 | 11 | 6 | 18 | 9 | 7 | 8 | 9 | 11 | 96 | 8 |
| 1956 | EGFR | 9 | 17 | 12 | 9 | 12 | 10 | 9 | 9 | 10 | 6 | 103 | 9 |
| 10987 | COPS5 | 11 | 12 | 15 | 11 | 10 | 11 | 11 | 12 | 12 | 9 | 114 | 10 |

Supplementary Table S4: Top 20% enriched GO terms after removal of the redundant terms.

| **S.No.** | **Term ID** | **Description** | **Process** | **Enrichment Score** |
| --- | --- | --- | --- | --- |
| 1 | GO:0031340 | positive regulation of vesicle fusion | BP | 12.85 |
| 2 | GO:0043353 | enucleate erythrocyte differentiation | BP | 12.85 |
| 3 | GO:1900084 | regulation of peptidyl-tyrosine autophosphorylation | BP | 12.85 |
| 4 | GO:2000366 | positive regulation of STAT protein import into nucleus | BP | 12.85 |
| 5 | GO:0015671 | oxygen transport | BP | 10.28 |
| 6 | GO:0031424 | keratinization | BP | 10.28 |
| 7 | GO:0051045 | negative regulation of membrane protein ectodomain proteolysis | BP | 10.28 |
| 8 | GO:0001895 | retina homeostasis | BP | 9.34 |
| 9 | GO:0060693 | regulation of branching involved in salivary gland morphogenesis | BP | 9.18 |
| 10 | GO:0015669 | gas transport | BP | 8.57 |
| 11 | GO:0043369 | CD4-positive or CD8-positive, alpha-beta T cell lineage commitment | BP | 8.57 |
| 12 | GO:0045109 | intermediate filament organization | BP | 8.57 |
| 13 | GO:0060644 | mammary gland epithelial cell differentiation | BP | 8.57 |
| 14 | GO:2001028 | positive regulation of endothelial cell chemotaxis | BP | 8.57 |
| 15 | GO:0031640 | killing of cells of other organism | BP | 8.03 |
| 16 | GO:0018149 | peptide cross-linking | BP | 7.71 |
| 17 | GO:0022612 | gland morphogenesis | BP | 7.34 |
| 18 | GO:0001836 | release of cytochrome c from mitochondria | BP | 7.23 |
| 19 | GO:0002251 | organ or tissue specific immune response | BP | 7.14 |
| 20 | GO:0002360 | T cell lineage commitment | BP | 7.14 |
| 21 | GO:0030214 | hyaluronan catabolic process | BP | 7.14 |
| 22 | GO:0043217 | myelin maintenance | BP | 7.14 |
| 23 | GO:0048552 | regulation of metalloenzyme activity | BP | 7.14 |
| 24 | GO:0051593 | response to folic acid | BP | 7.14 |
| 25 | GO:0070230 | positive regulation of lymphocyte apoptotic process | BP | 7.14 |
| 26 | GO:0031581 | hemidesmosome assembly | BP | 7.01 |
| 27 | GO:0042730 | fibrinolysis | BP | 7.01 |
| 28 | GO:0045103 | intermediate filament-based process | BP | 6.8 |
| 29 | GO:0001906 | cell killing | BP | 6.42 |
| 30 | GO:0010224 | response to UV-B | BP | 6.42 |
| 31 | GO:0032069 | regulation of nuclease activity | BP | 6.42 |
| 32 | GO:0032461 | positive regulation of protein oligomerization | BP | 6.42 |
| 33 | GO:0042832 | defense response to protozoan | BP | 6.42 |
| 34 | GO:0090344 | negative regulation of cell aging | BP | 6.42 |
| 35 | GO:1902230 | negative regulation of intrinsic apoptotic signaling pathway in response to DNA damage | BP | 6.09 |
| 36 | GO:0042744 | hydrogen peroxide catabolic process | BP | 6 |
| 37 | GO:0051412 | response to corticosterone | BP | 6 |
| 38 | GO:2001241 | positive regulation of extrinsic apoptotic signaling pathway in absence of ligand | BP | 5.93 |
| 39 | GO:0001562 | response to protozoan | BP | 5.84 |
| 40 | GO:0050832 | defense response to fungus | BP | 5.84 |
| 41 | GO:1903427 | negative regulation of reactive oxygen species biosynthetic process | BP | 5.84 |
| 42 | GO:0042743 | hydrogen peroxide metabolic process | BP | 5.51 |
| 43 | GO:0045987 | positive regulation of smooth muscle contraction | BP | 5.51 |
| 44 | GO:0050829 | defense response to Gram-negative bacterium | BP | 5.51 |
| 45 | GO:0050918 | positive chemotaxis | BP | 5.51 |
| 46 | GO:0061037 | negative regulation of cartilage development | BP | 5.51 |
| 47 | GO:2000811 | negative regulation of anoikis | BP | 5.51 |
| 48 | GO:2000352 | negative regulation of endothelial cell apoptotic process | BP | 5.29 |
| 49 | GO:0002576 | platelet degranulation | BP | 5.28 |
| 50 | GO:0002701 | negative regulation of production of molecular mediator of immune response | BP | 5.14 |
| 51 | GO:0009648 | photoperiodism | BP | 5.14 |
| 52 | GO:0034502 | protein localization to chromosome | BP | 5.14 |
| 53 | GO:0036003 | positive regulation of transcription from RNA polymerase II promoter in response to stress | BP | 5.14 |
| 54 | GO:0045429 | positive regulation of nitric oxide biosynthetic process | BP | 5.14 |
| 55 | GO:0071364 | cellular response to epidermal growth factor stimulus | BP | 5.14 |
| 56 | GO:1901797 | negative regulation of signal transduction by p53 class mediator | BP | 5.14 |
| 57 | GO:0010939 | regulation of necrotic cell death | BP | 5 |
| 58 | GO:0051881 | regulation of mitochondrial membrane potential | BP | 5 |
| 59 | GO:0045646 | regulation of erythrocyte differentiation | BP | 4.94 |
| 60 | GO:0048146 | positive regulation of fibroblast proliferation | BP | 4.86 |
| 61 | GO:0000083 | regulation of transcription involved in G1/S transition of mitotic cell cycle | BP | 4.82 |
| 62 | GO:0002449 | lymphocyte mediated immunity | BP | 4.82 |
| 63 | GO:0006984 | ER-nucleus signaling pathway | BP | 4.82 |
| 64 | GO:0010831 | positive regulation of myotube differentiation | BP | 4.82 |
| 65 | GO:0032459 | regulation of protein oligomerization | BP | 4.82 |
| 66 | GO:0032770 | positive regulation of monooxygenase activity | BP | 4.82 |
| 67 | GO:0022617 | extracellular matrix disassembly | BP | 4.74 |
| 68 | GO:0042100 | B cell proliferation | BP | 4.73 |
| 69 | GO:0090184 | positive regulation of kidney development | BP | 4.67 |
| 70 | GO:0043154 | negative regulation of cysteine-type endopeptidase activity involved in apoptotic process | BP | 4.61 |
| 71 | GO:0045840 | positive regulation of mitosis | BP | 4.56 |
| 72 | GO:0051155 | positive regulation of striated muscle cell differentiation | BP | 4.56 |
| 73 | GO:0048145 | regulation of fibroblast proliferation | BP | 4.55 |
| 74 | GO:0035987 | endodermal cell differentiation | BP | 4.5 |
| 75 | GO:1900408 | negative regulation of cellular response to oxidative stress | BP | 4.5 |
| 76 | GO:1903202 | negative regulation of oxidative stress-induced cell death | BP | 4.5 |
| 77 | GO:0045668 | negative regulation of osteoblast differentiation | BP | 4.47 |
| 78 | GO:0032642 | regulation of chemokine production | BP | 4.43 |
| 79 | GO:0032963 | collagen metabolic process | BP | 4.38 |
| 80 | GO:0031952 | regulation of protein autophosphorylation | BP | 4.28 |
| 81 | GO:0045599 | negative regulation of fat cell differentiation | BP | 4.28 |
| 82 | GO:1900046 | regulation of hemostasis | BP | 4.28 |
| 83 | GO:0042060 | wound healing | BP | 4.2 |
| 84 | GO:0044236 | multicellular organismal metabolic process | BP | 4.2 |
| 85 | GO:0060688 | regulation of morphogenesis of a branching structure | BP | 4.16 |
| 86 | GO:0031983 | vesicle lumen | CC | 5.05 |
| 87 | GO:0001533 | cornified envelope | CC | 12.85 |
| 88 | GO:0045095 | keratin filament | CC | 10.87 |
| 89 | GO:0002934 | desmosome organization | CC | 8.57 |
| 90 | GO:0030057 | desmosome | CC | 8.03 |
| 91 | GO:0031093 | platelet alpha granule lumen | CC | 5.71 |
| 92 | GO:0030863 | cortical cytoskeleton | CC | 5.65 |
| 93 | GO:0072562 | blood microparticle | CC | 5.4 |
| 94 | GO:0000307 | cyclin-dependent protein kinase holoenzyme complex | CC | 5.14 |
| 95 | GO:0008379 | thioredoxin peroxidase activity | MF | 12.85 |
| 96 | GO:0008191 | metalloendopeptidase inhibitor activity | MF | 8.57 |
| 97 | GO:0005172 | vascular endothelial growth factor receptor binding | MF | 8.57 |
| 98 | GO:0048551 | metalloenzyme inhibitor activity | MF | 8.57 |
| 99 | GO:0050786 | RAGE receptor binding | MF | 7.14 |
| 100 | GO:0005161 | platelet-derived growth factor receptor binding | MF | 6.42 |
| 101 | GO:0005200 | structural constituent of cytoskeleton | MF | 6.12 |
| 102 | GO:0042056 | chemoattractant activity | MF | 6 |
| 103 | GO:0030332 | cyclin binding | MF | 5.84 |

Supplementary Table S5: Status and the mRNA expression of the 39 predicted genes in Oncomine and expression Atlas.

| **Rank** | **Gene ID** | **Gene Symbol** | **Up/**  **Down** | **Log_2_FC^*^** | **P-value** | **Sample(N,C)^#^** | | **Data type** | **Reference(PMID)** | |
| --- | --- | --- | --- | --- | --- | --- | --- | --- | --- | --- |
| 1 | 3320 | HSP90AA1 | up | 2.55 | 2.98E-07 | 9,15 | | Tongue Carcinoma vs. Normal | 17510386 | |
| 1 | 3320 | HSP90AA1 | up | 3.01 | 1.40E-08 | 9,5 | | Floor of the Mouth Carcinoma vs. Normal | 17510386 | |
| 1 | 3320 | HSP90AA1 | up | 3.88 | 6.57E-09 | 9,4 | | Oral Cavity Carcinoma vs. Normal | 17510386 | |
| 3 | 2099 | ESR1 | down | -3.59 | 3.20E-04 | 9,4 | | Oral Cavity Carcinoma vs. Normal | 17510386 | |
| 4 | 3312 | HSPA8 | up | 5.87 | 1.81E-07 | 9,4 | | Oral Cavity Carcinoma vs. Normal | 17510386 | |
| 4 | 3312 | HSPA8 | up | 3.92 | 5.58E-05 | 9,5 | | Floor of the Mouth Carcinoma vs. Normal | 17510386 | |
| 4 | 3312 | HSPA8 | up | 3.95 | 2.32E-06 | 9,15 | | Tongue Carcinoma vs. Normal | 17510386 | |
| 8 | 5071 | PARK2 | down | -2.98 | 5.02E-06 | 26,31 | | Tongue Squamous Cell Carcinoma vs. Normal | 19138406 | |
| 13 | 1457 | CSNK2A1 | up | 2.31 | 1.69E-04 | 9,5 | | Floor of the Mouth Carcinoma vs. Normal | 17510386 | |
| 14 | 7013 | TERF1 | up | 2.25 | 1.41E-09 | 9,15 | | Tongue Carcinoma vs. Normal | 17510386 | |
| 14 | 7013 | TERF1 | up | 2.58 | 4.01E-09 | 9,5 | | Floor of the Mouth Carcinoma vs. Normal | 17510386 | |
| 14 | 7013 | TERF1 | up | 2.14 | 2.40E-04 | 9,4 | | Oral Cavity Carcinoma vs. Normal | 17510386 | |
| 15 | 5591 | PRKDC | up | 2.25 | 2.68E-13 | 26,31 | | Tongue Squamous Cell Carcinoma vs. Normal | 15833835 | |
| 15 | 5591 | PRKDC | up | 2.56 | 2.65E-11 | 26,31 | | Tongue Squamous Cell Carcinoma vs. Normal | 19138406 | |
| 16 | 5591 | PRKDC | up | 3.50 | 8.61E-07 | 9,15 | | Tongue Carcinoma vs. Normal | 17510386 | |
| 16 | 5591 | PRKDC | up | 7.30 | 2.29E-06 | 9,5 | | Floor of the Mouth Carcinoma vs. Normal | 17510386 | |
| 21 | 4176 | MCM7 | up | 3.47 | 6.02E-07 | 9,4 | | Oral Cavity Carcinoma vs. Normal | 17510386 | |
| 21 | 4176 | MCM7 | up | 5.94 | 2.47E-05 | 9,5 | | Floor of the Mouth Carcinoma vs. Normal | 17510386 | |
| 22 | 3676 | ITGA4 | up | 2.00 | 7.76E-08 | 22,57 | | Oral Cavity Squamous Cell Carcinoma vs. Normal | 21853135 | |
| 27 | 5515 | PPP2CA | up | 2.75 | 1.35E-05 | 9,5 | | Floor of the Mouth Carcinoma vs. Normal | 17510386 | |
| 28 | 2316 | FLNA | up | 2.93 | 7.80E-09 | 26,31 | | Tongue Squamous Cell Carcinoma vs. Normal | 19138406 | |
| 32 | 5747 | PTK2 | up | 2.82 | 4.93E-08 | 9,15 | | Tongue Carcinoma vs. Normal | 17510386 | |
| 32 | 5747 | PTK2 | up | 3.16 | 3.20E-04 | 9,5 | | Floor of the Mouth Carcinoma vs. Normal | 17510386 | |
| 37 | 998 | CDC42 | up | 2.22 | 1.76E-19 | 22,57 | | Oral Cavity Squamous Cell Carcinoma vs. Normal | 21853135 | |
| 37 | 998 | CDC42 | up | 2.51 | 1.81E-06 | 9,15 | | Tongue Carcinoma vs. Normal | 17510386 | |
| **Status and the mRNA expression of the 39 predicted genes in Expression Atlas** | | | | | | | | | | |
| **Rank** | **Gene ID** | **Gene Symbol** | **Up/Down** | **Log_2_FC^*^** | **P-value** | **Sample**  **(cancer, Normal)** | **Data type** | | | **Reference**  **(PMID)** |
| 3 | 2099 | ESR1 | up | 2.7 | 1.65E-22 | SCC-9, OKF6-TERT1R | tongue squamous cell carcinoma vs normal in vehicle | | | 24076275 |
| 4 | 3312 | HSPA8 | up | 1.3 | 0.002242 | SCC-9, OKF6-TERT1R | tongue squamous cell carcinoma vs normal in vehicle | | | 24076275 |
| 7 | 4088 | SMAD3 | up | 2.7 | 1.65E-22 | SCC-9, OKF6-TERT1R | tongue squamous cell carcinoma vs normal in vehicle | | | 24076275 |
| 8 | 5071 | PARK2 | down | -2.5 | 0.000115 | SCC-9, OKF6-TERT1R | tongue squamous cell carcinoma vs normal in vehicle | | | 24076275 |
| **28** | 2316 | FLNA | up | 1.3 | 6.86E-20 | A-AFFY-44 | tongue squamous cell carcinoma vs normal | | | HG-U133_Plus_2 |
| **31** | 8826 | IQGAP1 | down | -1.2 | 3E-06 | SCC-9, OKF6-TERT1R | tongue squamous cell carcinoma vs normal in vehicle | | | 24076275 |
| **33** | 6657 | SOX2 | down | -1.5 | 4.19E-06 | A-AFFY-44 | tongue squamous cell carcinoma vs normal | | | HG-U133_Plus_2 |
| **39** | 3688 | ITGB1 | down | -1.4 | 1.24E-07 | SCC-9, OKF6-TERT1R | tongue squamous cell carcinoma vs normal in vehicle | | | 24076275 |

*FC=Fold Change, #Samples (N,C)=Samples(Normal, Cancerous)

**Supplementary Table S6: Literature information on predicted genes.**

| S. N. | Gene Name | Role/function | PMID: | Year |
| --- | --- | --- | --- | --- |
| 1 | HSP90AA1 | Up regulation (stress response) | 24112734 | 2013 |
| 2 | HSP90AA1 | Heat shock protein 90 inhibitor, 17-allylamino-17-demethoxygeldanamycin has a synergistic effect on X-rays, but not carbon-ion beams, on lethality in human oral squamous cell carcinoma cells | 22843619 | 2012 |
| 3 | HSPA8 | Up regulation (stress response) | 24112734 | 2013 |
| 4 | MCM7 | RT-PCR and immunohistochemistry indicated positive expressions of Mcm7 mrna and protein in normal oral mucosa, precancerous lesions and oral squamous cell carcinoma. | 19189662 | 2008 |
| 5 | SMAD3 | The study reports SMAD3 mutations in oral carcinoma | 23913824 | 2013 |
| 6 | AKT1 | Physcion 8-O-β-glucopyranoside induces mitochondria-dependent apoptosis of human OSCC cells by suppressing survivin expression via mir-21/PTEN/Akt/GSK3β signaling pathway | 27063218 | 2016 |
| 7 | AKT1 | Study confirms the contribution of loss of PTEN expression in Akt phosphorylation and spontaneous apoptosis suppression in the specimens of oral cancer | 22033727 | 2012 |
| 8 | AKT1 | Expression levels of EGFR, pakt, and PTEN differ between oropharyngeal and oral cavity cancer and it may be attributed to HPV-related molecular pathogenesis | 22682934 | 2012 |
| 9 | AKT1 | Collectively, these data suggested that, in oral epithelial cells, the herpes simplex virus 1-induced PI3K/Akt activation was involved in the regulation of apoptosis blockage and viral gene expression. | 20620179 | 2010 |
| 10 | AKT1 | Results suggested that PTEN/AKT pathway was involved in trichostatin A induced cell growth inhibition and apoptosis of oral squamous cell carcinoma cells | 19574087 | 2009 |
| 11 | CTNNB1, AKT1 | AKT1 is slightly down regulated and CTNNB1 were upregulated in young patience of squamous cell carcinoma of tongue. | 25633809 | 2015 |
| 12 | CTNNB1 | High CTNNB1 expression is associated with oral squamous cell carcinoma. | 24083714 | 2013 |
| 13 | CTNNB1 | Studied association between beta-catenin expression and clinical-pathological parameters in 374 osccs/OP-sccs by immunohistochemistry (IHC). | 24511551 | 2014 |
| 14 | CTNNB1 | Loss of E-cadherin and beta-catenin with cytoplasmic ALCAM accumulation may play pivotal role in oral cancer development and progression. | 23840677 | 2013 |
| 15 | CTNNB1 | Expression and subcellular localization of p120-catenin and beta-catenin in oral carcinomas | 23936352 | 2013 |
| 16 | CTNNB1 | Beta-catenin and deltanp63 may be used as independent prognostic markers of oral carcinoma; interaction of beta-catenin with deltanp63 may be crucial event in regulating proliferation and differentiation of oral carcinoma cells; target for therapeuti | 21881870 | 2012 |
| 17 | CTNNB1 | Nuclear translocation of beta-catenin synchronized with loss of E-cadherin in oral epithelial dysplasia with a characteristic two-phase appearance. | 21884207 | 2011 |
| 18 | CTNNB1 | Loss of heterozygosity contributes to tumor progression of oral squamous cell carcinoma, and a specific role for PDCD4, CTNNB1, and CASP4 was found. | 21781452 | 2011 |
| 19 | CTNNB1 | The combination of the upregulation of vimentin and aberrant expression of E-cadherin/beta-catenin complexes at the tumour invasive front may provide a useful prognostic marker in oral squamous cell carcinoma. | 19915524 | 2010 |
| 20 | CTNNB1 | EGFR signaling regulates beta-catenin localization and stability, target gene expression, and tumor progression in oral cancer. | 20302655 | 2010 |
| 21 | CTNNB1 | The pathway including S100A7/psoriasin and beta-catenin signaling has a role in tumor progression of squamous cell carcinoma of oral cavity | 18223693 | 2008 |
| 22 | CTNNB1 | Data indicate that altered expression of beta-catenin may play an important role in oral cancer progression through increased proliferation and invasiveness under epidermal growth factor receptor (EGFR) activation but not mutation or cyclin D1 expression | 15791567 | 2005 |
| 23 | CTNNB1 | Wnt-1, beta-catenin and APC expressions were related to the differentiation of oral squamous cell carcinoma. | 16329837 | 2005 |
| 24 | CTNNB1 | The cytoplasmic accumulation of beta-catenin is a common characteristic of oral SCC, but is not closely associated with mutational alterations in the APC, beta-catenin and Axin1 genes. | 16163548 | 2005 |
| 25 | CTNNB1 | The reduced immunoexpression of beta-catenin in the membrane may be related to the high degree of cell indifferentiation in cases of oral squamous cell carcinoma with high scores. | 19118778 | 2001 |
| 26 | CDC42 | Snail regulates the motility and invasiveness of oral cancer cells via rhoa/Cdc42/p-ERM pathway. | 25172658 | 2014 |
| 27 | ESR1 | Oral squamous cell carcinoma cells expressed functional era whose Ser118 phosphorylation and transcriptional activity were enhanced by FAK/AKT signaling, leading to promoted cell growth | 24825747 | 2014 |
| 28 | RELA | Our results indicate that cetuximab may enhance the effect of PTX in OSCC through the downregulation of PTX induced p65 expression  Immunohistochemical staining revealed that expression of p65 was downregulated in HSC2 tumors treated with PTX and cetuximab | 25230791 | 2014 |
| 29 | RELA | Results show that Id1 and NF-kappab regulate the expression of CD133 and BMI-1 in an additive or synergistic manner in oral squamous cell carcinoma. | 24572994 | 2014 |
| 30 | RELA | IL-23 up-regulates the growth and cell proliferation of oral cancer by promoting the nuclear transactivation of rela | 20428758 | 2010 |
| 31 | SRC | Src family kinases mediate betel quid-induced oral cancer cell motility | 19048118 | 2008 |
| 32 | SRC | p130Cas, Src and talin function in both oral carcinoma invasion and resistance to cisplatin | 21291860 | 2011 |
| 33 | SRC | The expression of Src protein is significantly associated with the progression, recurrence, and prognosis of oral squamous cell carcinoma in Taiwan. | 22052839 | 2012 |
| 34 | NOTCH1 | These results suggest that NOTCH1 mutation occurs frequently in Japanese OSCC in the vicinity of the ligand binding region and, these mutations cause downregulation of the NOTCH1 function. | 25234595 | 2014 |
| 35 | NOTCH1 | Data suggest Notch1/NICD (intracellular Notch domain) are up-regulated at invasive tumor front in oral squamous cell carcinoma; Notch1 expression correlates with pathological stage, clinical stage, invasiveness, proliferation, and perhaps metastasis. |  |  |
| 36 | NOTCH1 | Notch1 mutation is common in Chinese oral squamous cell carcinoma and associates with clinical outcomes | 24277457 | 2014 |
| 37 | NOTCH1 | Downregulated expression of Notch 1 was related to invasion and differentiation status of oral carcinoma cells. | 21881870 | 2012 |
| 38 | PTK2 | FAK was highly expressed in oral squamous cell carcinoma tissues, in contrast to none or a low expression in normal oral epithelial tissue, and the heterogeneous staining was mainly located in the cell membrane and cytoplasm | 17236584 | 2006 |
| 39 | PTK2 | These results suggest that Bim, Bcl-xl, FAK and endonuclease G are involved in safingol-induced apoptosis of detached oral squamous cell carcinoma. | 19199036 | 2009 |
| 40 | PTK2 | Our results support the hypothesis that FAK activity might be involved in the down-regulation of p53 expression in oral squamous cell carcinoma | 22790665 | 2012 |
| 41 | RAF1 | Studied the promoter methylation and RNA expression profiles of ERK and RAF1 genes with risk of oral squamous cell carcinoma (OSCC) along with the promoter methylation status of ERK and RAF1 genes. | 25265753 | 2014 |
| 42 | SOX2 | The results indicate that high expression rates of SOX2 might be a prediction marker for oral squamous cell carcinoma lymph node metastasis. | 23005595 | 2012 |
| 43 | SOX2 | Sox2 positive expression was frequent in pn0 OTSCC and involved in tumor progression. | 21689966 | 2011 |
| 44 | SOX2 | Data indicate that sex determining region Y (SRY)-box 2 (SOX2) up-regulation is frequent in early squamous cell carcinomas of the oral cavity and associated with decreased risk of lymphatic metastasis. | 23414798 | 2013 |
| 45 | SOX2 | Oct4+Sox2+ profile may contribute to the malignant transformation of oral mucosa | 24427323 | 2013 |
| 46 | ITGA4 | ITGA4 knockdown decreased p38 phosphorylation and increased apoptosis | 23511560 | 2013 |
| 47 | ITGA4 | FN1 and ITGA4 are potential oral squamous cell carcinoma biomarkers for tongue/mouth floor and edentulous ridge | 23116545 | 2013 |
| 48 | CDC42 | These results indicate that Wnt5b is involved in the migration ability of OSCC cells through active Cdc42 and rhoa | 24220306 | 2014 |
| 49 | FLNA | Upregulation in oscc by Westron,ihc MALDI-TOF/TOF | 19297561 | 2009 |
| 50 | FLNA | Downregulation MALDI-TOF/TOF | 25295583 | 2015 |
| 51 | FLNA | 4 genes (SPCS1, FLNA, CHPF and  GLT8D1) had significantly upregulated mrna expression levels in  HNSCC tumours. FLNA (filamin A, alpha), an actin-binding  Protein involves in cytoskeletal/membrane remodelling and  Cellular motility | 2461910 | 2012 |
| 52 | HSPA8 | These findings suggest that histatin 3 may be involved in cell proliferation through the regulation of HSC70 and p27(Kip1) in oral cells. | 19321452 | 2009 |
| 53 | MAPK14 | EGF and HGF induced cell migration in human oral squamous carcinoma cell lines was dependent on the MEK ERK, the p38 and the PI-3 kinase pathways for both growth factors. | 22413835 |  |
| 54 | CALM1, 2, 3, FLNA, HSP90AA1, HSPA8, IQGAP1 | Proteins were differentially expressed in global proteomic analysis of oral cavity squamous cell carcinoma using imaging mass spectrometry | 25295583 | 2015 |
| 55 | CTNNB1, NOTCH1, SRC, SMAD3 | Predicted by PPI network analysis of OSCC related genes using nearest neighbor expansion method | 24083714 | 2013 |
| 56 | SIRT1 | Demonstrated that overexpression of Sirt1 survived OSCC cell line Tca8113 under cisplatin treatment | 21947960 | 2011 |
| 57 | SIRT1 | Multivariate analyses revealed that expression of SIRT1 was an independent and good indicator of prognosis. | 23453030 | 2013 |
| 58 | SIRT1 | Antitumor effect in oral squamous cell carcinoma cells is related to the activation of MAPK/and NF-κb as well as of the SIRT1 pathway | 23881456 | 2013 |
| 59 | JAK2 | This apoptosis induction was associated with marked suppression of JAK2/STAT3, Akt and Erk signaling pathways in honokiol-treated SAS spheres. | 27012679 | 2016 |

**Supplementary Table S7: Mean expression index of each grade and TANT.**

| **Gene** | **TANT** | **Grade 1** | **Grade 2** | **Grade 3** |
| --- | --- | --- | --- | --- |
| **FLNA** | **1.5** | 2.39 | 1.67 | 1.6 |
| **ARRB1** | **1.4** | 0.87 | 1.00 | 0.8 |
| **HTT** | **2.2** | 1.95 | 1.67 | 1.8 |
| **CALM3** | **2.4** | 2.29 | 2.33 | 2.8 |

**Supplementary Table S8: The centrality scores for selected genes.**

| **Gene ID** | **408** | **3064** | **2316** | **808** | **Avg. Centrality** |
| --- | --- | --- | --- | --- | --- |
| **Gene Symbol** | **ARRB1** | **HTT** | **FLNA** | **CALM3** |  |
| **Degree** | 144 | 83 | 83 | 144 | 17.42 |
| **Closeness** | 0.40 | 0.41 | 0.41 | 0.40 | 0.32 |
| **Centroid** | -1530 | -1365 | -1365.00 | -1530 | -3804.61 |
| **SPB** | 91882.20 | 36981.54 | 36981.54 | 91882.20 | 5112.48 |
| **Eigenvector** | 0.05 | 0.05 | 0.05 | 0.05 | 0.01 |
| **page rank** | 0.002 | 0.001 | 0.001 | 0.002 | 0.000213 |
| **CFB** | 3.03 | 2.98 | 2.98 | 3.03 | 1.95 |
| **CFC** | 0.01 | 0.01 | 0.01 | 0.01 | 0.00 |
| **Stress** | 3988108.00 | 1847366.00 | 1847366 | 3988108.00 | 216168.70 |
| **vulnerability** | 0.002 | 0.001 | 0.001 | 0.002 | 0.000509 |
| **All AVG Rank** | **48** | **59** | **128** | **96** |  |


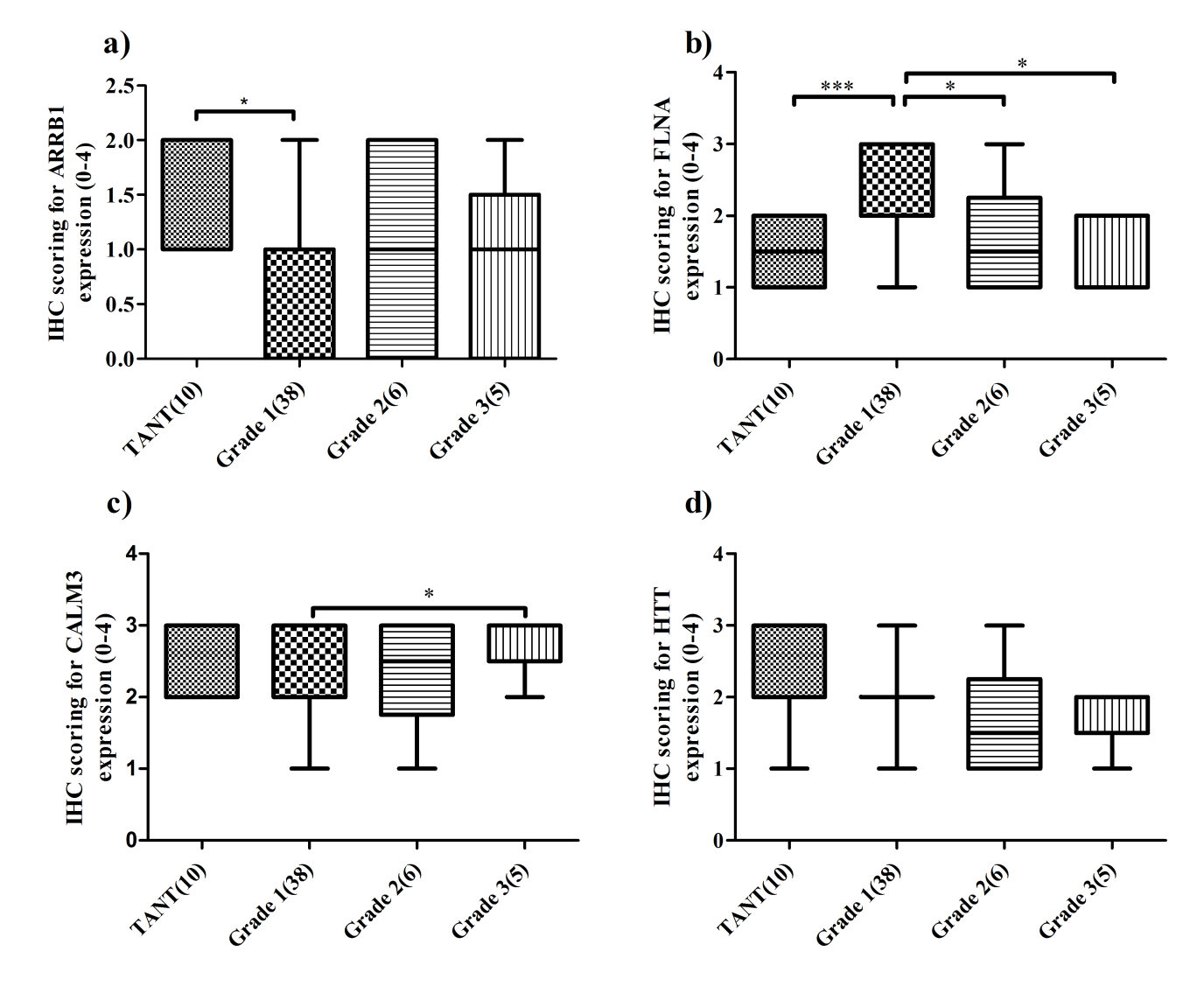


**Supplementary Figure S1:** Supplementary Figure S1: Box plot showing grade wise expression of the genes on immunohistochemically stained TMA sections of OSCC samples. The scoring was done on a scale of 0 to 4 [where 0: no staining, 1: 25% (mild staining), 2: 25–50% (medium staining), 3: 50–75% (moderate staining) and 4: ≥75% (strong staining)]. a) ARRB1 expression was found to be decreased significantly (p=0.033) from TANT to cancerous grade 1 samples but the change was not significant within the grades b) Significantly upregulated expression of FLNA was found in all grades of the oral cancer samples as compared to the TANT (p<0.05) but within the grades (grade 1 to grade 3) the expression goes down significantly (p<0.05). c) The expression of CALM3 did not show significant change from TANT to grade 1 cancerous samples but between grade 1 and grade 3 the expression is significantly upregulated (p=0.041). d) HTT showed a decreased expression from TANT to cancerous sample but the decrease is not significant. The number of samples used is indicated in brackets.


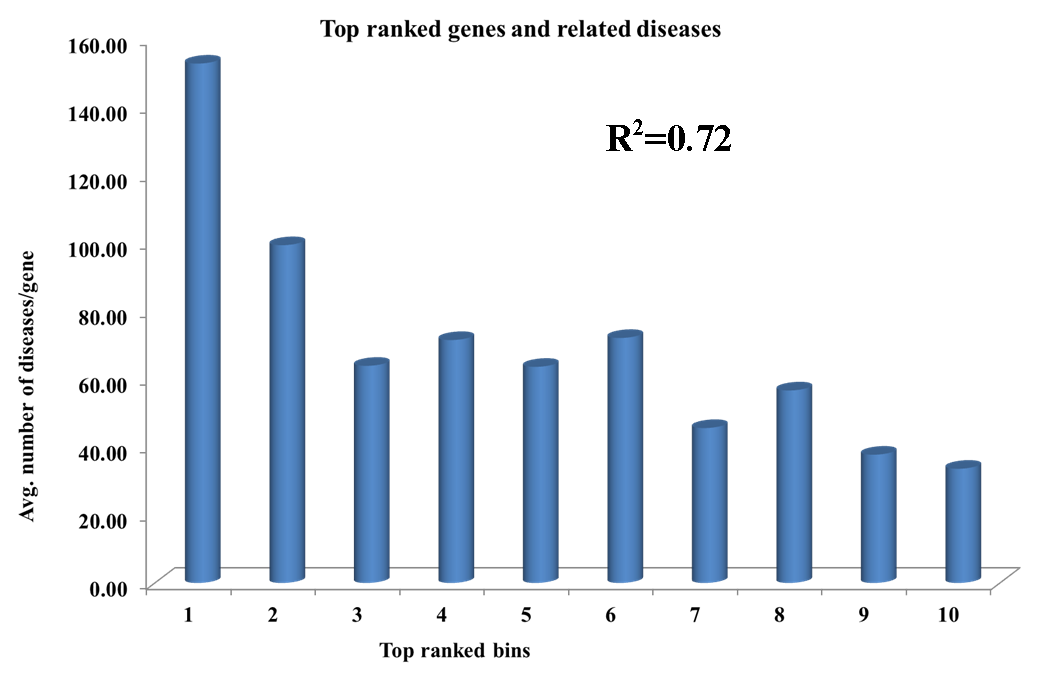


**Supplementary Figure S2:** The HCGN genes (total 4704) were distributed into ten equally sized bins as per consensus rank. Average number of diseases/genes was extracted from DisGeNET all disease-gene association database for each group. X- Axis represents a bin number from 1 to 10 whereas Y-axis represents the average number of disease/gene corresponding to each bin.

**References**

1 Junker, B. H., Koschutzki, D. & Schreiber, F. Exploration of biological network centralities with CentiBiN. *BMC bioinformatics* **7**, 219, doi:10.1186/1471-2105-7-219 (2006).

2 Brandes, U., Erlebach, T. & SpringerLink (Online service). in *Lecture notes in computer science,* (Springer-Verlag GmbH,, Berlin Heidelberg, 2005).

3 Brandes, U., Erlebach, T. & SpringerLink (Online service). in *Network Analysis Methodological Foundations* (ed V. Diekert and B. Durand) 533–544 (Springer-Verlag GmbH,, 2005).

4 Koschutzki, D. & Schreiber, F. Centrality analysis methods for biological networks and their application to gene regulatory networks. *Gene regulation and systems biology* **2**, 193-201 (2008).

5 Koschutzki, D. Centralities in Biological Networks. (2006).

6 Scardoni, G., Petterlini, M. & Laudanna, C. Analyzing biological network parameters with CentiScaPe. *Bioinformatics* **25**, 2857-2859, doi:10.1093/bioinformatics/btp517 (2009).

7 Hindumathi, V., Kranthi, T., Rao, S. B. & Manimaran, P. The prediction of candidate genes for cervix related cancer through gene ontology and graph theoretical approach. *Molecular bioSystems* **10**, 1450-1460, doi:10.1039/c4mb00004h (2014).
